# *In Vivo* Effects of Histone H3 Depletion on Nucleosome Occupancy and Position in *Saccharomyces cerevisiae*

**DOI:** 10.1371/journal.pgen.1002771

**Published:** 2012-06-21

**Authors:** Andrea J. Gossett, Jason D. Lieb

**Affiliations:** 1 Department of Biology, Carolina Center for Genome Sciences, The University of North Carolina at Chapel Hill, Chapel Hill, North Carolina, United States of America; 2 Lineberger Comprehensive Cancer Center, The University of North Carolina at Chapel Hill, Chapel Hill, North Carolina, United States of America; University of Massachusetts Medical School, United States of America

## Abstract

Previous studies in *Saccharomyces cerevisiae* established that depletion of histone H4 results in the genome-wide transcriptional de-repression of hundreds of genes. To probe the mechanism of this transcriptional de-repression, we depleted nucleosomes *in vivo* by conditional repression of histone H3 transcription. We then measured the resulting changes in transcription by RNA–seq and in chromatin organization by MNase–seq. This experiment also bears on the degree to which trans-acting factors and DNA–encoded elements affect nucleosome position and occupancy *in vivo*. We identified ∼60,000 nucleosomes genome wide, and we classified ∼2,000 as having preferentially reduced occupancy following H3 depletion and ∼350 as being preferentially retained. We found that the *in vivo* influence of DNA sequences that favor or disfavor nucleosome occupancy increases following histone H3 depletion, demonstrating that nucleosome density contributes to moderating the influence of DNA sequence on nucleosome formation *in vivo*. To identify factors important for influencing nucleosome occupancy and position, we compared our data to 40 existing whole-genome data sets. Factors associated with promoters, such as histone acetylation and H2A.z incorporation, were enriched at sites of nucleosome loss. Nucleosome retention was linked to stabilizing marks such as H3K36me2. Notably, the chromatin remodeler Isw2 was uniquely associated with retained occupancy and altered positioning, consistent with Isw2 stabilizing histone–DNA contacts and centering nucleosomes on available DNA *in vivo*. RNA–seq revealed a greater number of de-repressed genes (∼2,500) than previous studies, and these genes exhibited reduced nucleosome occupancy in their promoters. In summary, we identify factors likely to influence nucleosome stability under normal growth conditions and the specific genomic locations at which they act. We find that DNA–encoded nucleosome stability and chromatin composition dictate which nucleosomes will be lost under conditions of limiting histone protein and that this, in turn, governs which genes are susceptible to a loss of regulatory fidelity.

## Author Summary

Chromatin is formed by wrapping 146 bp of DNA around a disc-shaped complex of proteins called histones. These protein–DNA structures are known as nucleosomes. Nucleosomes help to regulate gene transcription, because nucleosomes compete with transcription factors for access to DNA. The precise positioning and level of nucleosome occupancy are known to be vital for transcriptional regulation, but the mechanisms that regulate the position and occupancy of nucleosomes are not fully understood. Recently, many studies have focused on the role of DNA sequence and chromatin remodeling proteins. Here, we manipulate the concentration of histone proteins in the cell to determine which nucleosomes are most susceptible to changes in occupancy and position. We find that the chromatin-associated proteins Sir2 and Tup1, and the chromatin remodelers Isw2 and Rsc8, are associated with stabilized nucleosomes. Histone acetylation and incorporation of the histone variant H2A.z are the factors most highly associated with destabilized nucleosomes. Certain DNA sequence properties also contribute to stability. The data identify factors likely to influence nucleosome stability and show a direct link between changes in chromatin and changes in transcription upon histone depletion.

## Introduction

Twenty-five years ago, Michael Grunstein's laboratory began a series of experiments in which histones H2B or H4 were depleted *in vivo* in *S. cerevisiae* (yeast). After histone gene silencing, the yeast cells complete a single round of DNA replication, reducing their histone-DNA ratio by a factor of approximately two, and enter cell-cycle arrest. A number of conditionally expressed genes, including *PHO5*, *GAL1*, *CYC1*, *CUP1*, and *HIS3*, were reported to be transcriptionally de-repressed following histone depletion [1]–[4]. In 1999, Richard Young's laboratory revisited the transcriptional effects of H4 depletion, this time on a genomic scale using microarrays. Roughly 15% (888) of yeast genes were de-repressed 3-fold or greater, and another 10% of genes (569) were repressed at least 3-fold following H4 depletion [5]. Southern blots revealed changes in chromatin structure upstream of the *PHO5* promoter during H4 depletion, but at the time these experiments were performed, high-resolution genomic methods were not available to measure the widespread chromatin changes hypothesized to underpin the observed transcriptional changes. We therefore revisited these classic experiments using RNA-seq and MNase digestion coupled with next-generation sequencing (MNase-seq) to monitor RNA and chromatin changes after histone depletion. These experiments also bear on the recent debate regarding the degree to which DNA-encoded elements or other cellular factors control nucleosome position and occupancy [6]–[10]. The raw RNA-seq and MNase-seq data are available at GEO accession number GSE29294.

## Results

### H3 depletion results in changes in nucleosome occupancy throughout the genome

**Figure 1:**
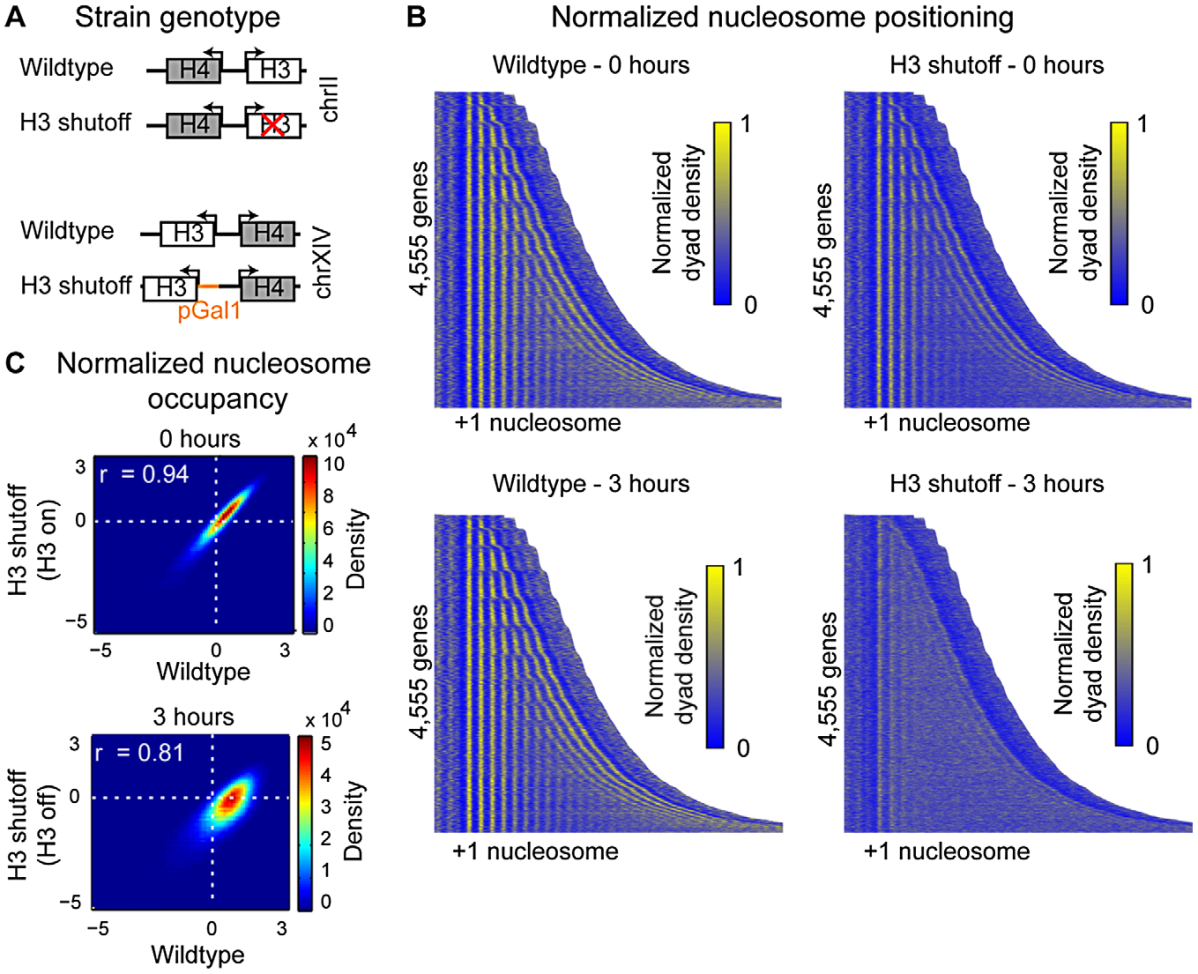
Histone H3 depletion alters nucleosome occupancy genome-wide. (A) Schematic representation of the strains used in this experiment. The H3 shutoff strain contains a deletion of one copy of histone H3 (*HHT1*) and a *GAL1* promoter inserted upstream of the second copy (*HHT2*). (B) The average, Gaussian-smoothed dyad density for the wildtype and H3 shutoff strains at 0 and 3 hours were aligned on the +1 nucleosomes for 4,555 genes. Upon H3 depletion, the regular array of nucleosomes visible in both of the wildtype time points and the 0 hour H3 shutoff strain time point become disrupted, resulting in a loss of regular positioning of nucleosomes internal to the gene. (C) Log_2_ normalized average nucleosome occupancy in the wildtype strain versus the H3 shutoff strain. Genome-wide nucleosome occupancy in the two strains is similar prior to H3 depletion (r = 0.94) but decreases following H3 depletion (r = 0.81).

Two genes encode histone H3 in wildtype *S. cerevisiae*, *HHT1* and *HHT2*. We obtained a strain in which *HHT1* had been deleted and *HHT2* had been placed under control of the *GAL1* promoter (Figure 1A) [11]. When grown in galactose, this “H3 shutoff” strain (DCB200.1) grows similarly to wildtype yeast (YEF473A), whereas cultivation in dextrose results in growth arrest in the G2/M phase of the cell cycle after a single round of DNA synthesis as large-budded cells (Figure S1A, S1B) [11]. After 3 hours in dextrose, RNA-seq shows that *HHT2* transcription is reduced to near zero, and Western blot analysis shows that histone H3 protein levels have dropped by a factor of two, as expected (Figure S1C, S1D). This evidence, coupled with previous cytological evidence using this strain [11], shows that the switch to dextrose successfully depleted histone H3.

We mapped nucleosome position and relative nucleosome occupancy using MNase-seq in the wildtype and H3 shutoff strains at 0 and 3 hours after transitioning the cells from galactose media to dextrose media (Figure S2A–S2C). While previous studies examined transcriptional changes 6 hours after the shift [5], we chose 3 hours for our study based on our characterization of the rate of histone depletion (Figure S1A–S1D) and to decrease the time the cells were under stress. Consistent with previous studies [2], [12], the chromatin was generally more sensitive to MNase digestion following H3 depletion, resulting in increased background smearing (Table S1, p<0.05; Figure S2D–S2G). Mono-nucleosome bands were extracted from the gel and sequenced, and the resulting reads were mapped back to the genome (Materials and Methods).

For the four possible strain and growth combinations, we aligned all of the genes by their +1 nucleosome relative to the transcription start site (TSS) [13] and calculated the smoothed dyad density across the gene body (Materials and Methods). The genes were then sorted by length from shortest to longest (Figure 1B). This plot reveals several key features of our dataset. First, the nucleosome organization of the wildtype strain in galactose (0 hours) is very similar to the nucleosome organization of the H3 shutoff strain in galactose (0 hours). Second, the nucleosome organization of the wildtype strain in galactose is very similar to the nucleosome organization of the wildtype strain in dextrose (3 hours). Third and most importantly, there are dramatic changes in nucleosome organization in the H3 shutoff strain between galactose (normal histone levels) and dextrose (depleted levels of H3). As histone availability decreases, the overall nucleosome positioning at any given position is weaker. Nucleosome positioning is still evident at the +1 and +2 nucleosomes but then decays rapidly, leading to a nearly complete loss of visible positioning until the gene's transcription termination site (TTS) is reached. There, one can still see a nucleosome at the −1 position relative to the TTS, although it is much more weakly positioned than in the wildtype strain.

To assess the overall similarity of our experimental replicates, we compared the overall nucleosome occupancy for each base pair among the replicates and found the experiments to be highly correlated (Figure S2H–S2K). As expected from the positioning data, the wildtype and H3 shutoff strains had very highly correlated nucleosome occupancy profiles when grown in galactose. When both strains were grown in dextrose, which results in histone H3 depletion in the shutoff strain, the correlation between the nucleosome occupancy profiles of the wildtype and H3 shutoff strains was lower, as expected (Figure 1C).

### Defined subsets of nucleosomes are susceptible to changes in occupancy and/or position following H3 depletion

**Figure 2:**
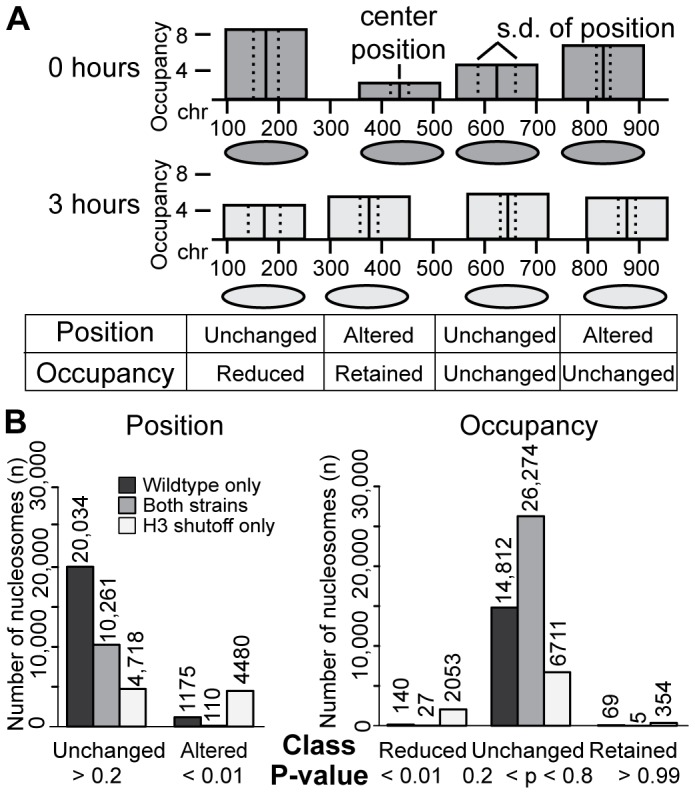
A subset of nucleosomes demonstrate behaviors specific to H3 depletion. (A) Schematic representation of the nucleosome classification method. Nucleosomes were classified based on changes in center positions and changes in read center counts between 0 hours and 3 hours (Materials and Methods). (B) Classification of nucleosomes. The number of nucleosomes in each classification that occur in only the wildtype strain 3 hours after the shift to Galactose (black); only the H3 shutoff strain (white); and those classified identically in both strains (gray) is shown. Only the H3 shutoff-specific nucleosomes (white) were used in subsequent analyses. The total number of nucleosomes that could be classified in each category are as follows: wildtype, 43,508; H3 shutoff, 38,955; classified in both, 28,717.

To identify changes in specific nucleosomes, we determined the position and occupancy of individual nucleosomes in each replicate at each time point (Materials and Methods). We used a slightly modified version of previously proposed definitions for measuring nucleosome positions and occupancies [14] that relies upon the midpoint of the paired-end reads or the center of the extended single-end reads (the “nucleosome dyad”) when examining nucleosome position and occupancy (Materials and Methods; Figure 2A). This method reduces positional noise that might be introduced from MNase producing fragments of varying lengths.

Changes in each nucleosome's position between the 0- and 3-hour time points were assessed using the t-test on the distribution of the read centers. The nucleosome position was then classified as being “altered” or “unchanged” based on the resulting p-value. Nucleosomes with borderline p-values were placed in a “no call” category and not used for the downstream analyses (Materials and Methods; Figure S3). The use of the t-test allowed us to account statistically for the “fuzziness” of each individual nucleosome. We note that p-values represent the confidence that an observation differs from the null expectation, not the degree to which an observation is biologically relevant.

Changes in each nucleosome's occupancy were assayed using the binomial distribution test on the number of read centers that fell within a 100-bp window centered on the nucleosome's consensus dyad. We stress that our experimental approach did not allow absolute nucleosome occupancy calculations, so the occupancy at a given location is in effect measured relative to all other nucleosomes in the same experiment. Nucleosomes were classified as “preferentially reduced” (decreased occupancy relative to other nucleosomes), “unchanged” (similar occupancy relative to others), or “preferentially retained” (increased relative occupancy). Nucleosomes with borderline p-values were placed in a separate “no call” category, similar to the position classification (Figure S3).

Nucleosomes that were classified identically in two or more replicates were selected as being “reliably characterized” (Materials and Methods; Figure S3). By comparing nucleosomes that were reliably characterized in both the wildtype and H3 shutoff experiment, we were able to remove nucleosomes for which the behavior could be attributed to the change in carbon source (Figure 2B and Figure S3). All further analysis was performed only with the “H3-depletion dependent” set of nucleosomes. The source code for all of the custom nucleosome calling tools can be found at http://sourceforge.net/p/callnucleosomes.

As an illustration of our nucleosome calls, we produced a graphical representation of the nucleosome structure surrounding *PHO5*, which had been previously shown to have altered chromatin and increased transcription following H3 depletion [1], and *IDP2*, a gene with increased transcription following H3 depletion (Figure S4 and Figure S5). After H3 shutoff, a new nucleosome configuration was observed upstream of both genes.

### Preferentially lost nucleosomes tend to occur at gene promoters and to be acetylated

**Figure 3:**
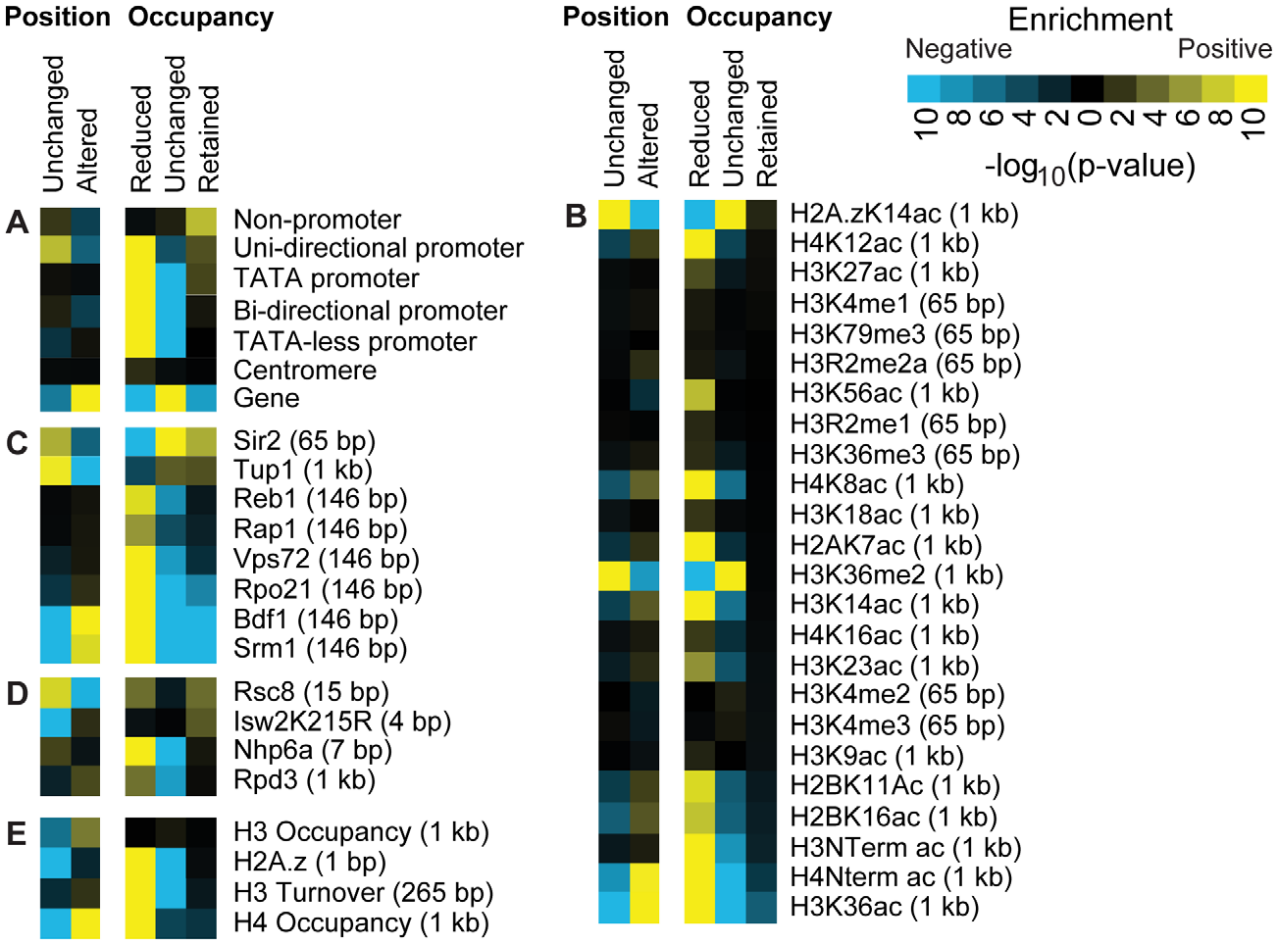
Histone acetylation is associated with reduced nucleosome occupancy. The positive (yellow) or negative (blue) association of previously published genome-wide data sets and genome annotations with nucleosome change categories (Materials and Methods). (A) Genome annotations, (B) histone modifications, (C) transcription-associated proteins, (D) chromatin remodelers and (E) histone occupancy. The number in parenthesis indicates the resolution of the detection platform in the data set used for comparison.

We sought to understand the factors that were responsible for the nucleosomes that were preferentially lost or retained or that responded to histone depletion by altering their positions. We compared each of the five classes of nucleosome changes (“position altered”, “position unchanged”, “occupancy reduced”, “occupancy unchanged”, and “occupancy retained”) to genome annotations and to previously published ChIP-chip or ChIP-seq experiments, including *in vivo* maps of histones, histone modifications, histone variants, and DNA-associated proteins (Figure 3 and Figure S6 and Table S1). Nearly all of the published data we used was obtained under standard growth conditions in dextrose, which matches the growth conditions of our strains upon the initiation of the H3 transcriptional shutoff (Materials and Methods). Therefore, the published datasets offer a reasonable representation of the chromatin landscape during histone depletion. We note that while our data has single-base pair technical resolution, the resolution of the published datasets varies (see Figure 3). Therefore statements about nucleosomes lacking or harboring a specific histone modification or other property should be interpreted with this in mind.

Upon histone H3 depletion, reduced-occupancy nucleosomes tended to occur in promoter regions, while nucleosomes in the gene bodies were associated with unchanged occupancy (Figure 3A). Consistent with this, a mark associated with transcriptional elongation, H3K36me2, was strongly enriched on nucleosomes classified as “position unchanged” or “occupancy unchanged” (Figure 3B). H3K36me2 has been proposed to contribute to increased nucleosome stability in the wake of RNA Polymerase II (RNA Pol II) transcription via the recruitment of the histone deacetylase (HDAC) Rpd3 [15], [16].

Consistent with nucleosome destabilization due to histone acetylation, “altered position” or “reduced occupancy” nucleosomes were enriched for 14 of the 16 of the histone acetylation marks we examined (p<0.01) (Figure 3B). Acetylated nucleosomes are generally less stable than those that are not acetylated [17]–[19], so it follows that acetylated nucleosomes would be more susceptible to changes following H3 depletion. We also found that nucleosomes with reduced occupancy or altered position tended to exhibit high replication-independent turnover rates (Figure 3E and Figure S7) [20], suggesting that the more dynamic histone-DNA interactions at these locations result in a relative loss under histone-limiting conditions.

### The conserved co-repressor Tup1 is associated with stable nucleosome positions and occupancy, while Bdf1, Rap1, and Reb1 binding are associated with nucleosome loss

We compared the H3-depletion responsive nucleosomes to previously published genome-wide data for several transcription factors, including Bdf1, Rap1, Reb1, and Tup1 [21], [22]. Sites of Bdf1, Rap1, and Reb1 binding were all associated with reduced nucleosome occupancy following H3 depletion, which is consistent with the known ability of these proteins to displace nucleosomes (Figure 3C).

In contrast, Tup1 is a known transcriptional repressor that can recruit histone deacetylases (HDACs) [23]–[26]. Sites of Tup1 binding were associated with “position unchanged” and “occupancy retained” or “occupancy unchanged” nucleosomes (Figure 3C). These results support the hypothesis that Tup1 represses transcription by stabilizing nucleosome position and occupancy [21], [27].

### Isw2 is associated with the unique combination of retained occupancy and altered position, providing evidence for its biochemical activity *in vivo*

Isw2 is an ATP-dependent chromatin remodeler that positions nucleosomes at the 5′ and 3′ ends of genes by binding to both the histone octamer and DNA [28], [29]. ISW2 is known to catalyze the centering of a nucleosome on a DNA substrate *in vitro* [30], [31]. Based on the distribution of catalytically inactive Isw2 enzyme (Isw2K215R) [32], Isw2 is associated with the unique combination of “occupancy retained” and “position altered” nucleosomes in our experiment. This is in contrast to most other factors associated with “occupancy retained” nucleosomes, which are typically classified as “position unchanged” nucleosomes (Figure 3D).

Based on its biochemical nucleosome-centering activity [30], [31], we hypothesized that Isw2 may help retain nucleosome occupancy by stabilizing histone-DNA interactions, while at the same time causing the position of bound nucleosomes to be especially sensitive to the loss of an adjacent nucleosome. Adjacent nucleosome loss could provide free DNA for the nucleosome centering activity of ISW2. Consistent with this hypothesis, of the 653 nucleosomes bound by Isw2K215R and classified as “position altered”, 429 move in the same direction in all 4 replicates, while another 142 shift in the same direction in 3 of the 4 replicates, indicating that for over 85% of the affected nucleosomes there is a clear directionality to the Isw2-associated shift *in vivo* (Figure S8A). The shift in position of these Isw2-bound nucleosomes was strongly associated with a decrease in nucleosome occupancy within 600 bp of the direction of the positional shift (p = 6.9E−11). In other words, the Isw2-bound nucleosomes consistently shifted specifically in the direction of a nearby, lost nucleosome. In contrast, Isw2-bound nucleosomes that do not change position are associated with adjacent “occupancy retained” nucleosomes (p = 0.059) and are not associated with a reduced occupancy of adjacent nucleosomes (p = 1), suggesting that a loss of adjacent nucleosomes is required for ISW2-mediated positional changes (Figure S8B, S8C). Fully consistent with the known *in vitro* activity of ISW2, the “linker” length (distance to the next mapped nucleosome) increased both upstream and downstream of Isw2-bound nucleosomes following nucleosome depletion such that the Isw2-bound nucleosomes became centered on the new local DNA substrate. While the ability of Isw2 to slide nucleosomes *in vivo* has been demonstrated previously [33], our result suggests that under conditions of lowered nucleosome density, Isw2 acts to create regularly-spaced nucleosomal arrays *in vivo*. We note that position-altered nucleosomes not bound by the mutant Isw2K215R as described in [32] were also centered, suggesting that wildtype Isw2 or another remodeling enzyme may center nucleosomes on newly-created gaps during genome-wide histone depletion (Figure S8D).

“Position altered,” Isw2-bound nucleosomes tended to contain the histone variant H2A.z (p<0.01) relative to all Isw2-bound nucleosomes [34], while Isw2-bound nucleosomes that were stably positioned were enriched for Tup1 (p<0.001) and Rsc8 (p<0.0001) binding relative to all Isw2-bound nucleosomes. Tup1 has previously been shown to localize independently to Isw2-bound regions, suggesting that the two proteins may work independently to maintain nucleosome position [35]. Taken together, these patterns suggest that Isw2's centering function *in vivo* may be aided by incorporation of H2A.z and restricted by Tup1 and Rsc8.

### Nhp6a is associated with preferential loss of nucleosomes specifically in transcribed regions

Nhp6a is an HMG-group protein known to associate with chromatin (reviewed in [36]). Loss of Nhp6a interferes with conditional gene activation [37] and has been reported to stabilize nucleosomes at promoters [38]. Human HMGB1 aids in depositing nucleosomes on a DNA template *in vitro* [6], and deletion of both Nhp6a and Nhp6b in *S. cerevisiae* results in a 20–30% decrease in histone levels *in vivo* [6], suggesting that Nhp6a/b functions in regulating nucleosome stability or deposition.

We found that previously measured Nhp6a binding was associated with nucleosomes that were preferentially lost in our experiments following H3 depletion (Figure 3D) [38]. This is generally consistent with a role for Nhp6 in nucleosome destabilization or deposition. To investigate if Nhp6a has different functions in promoters and gene bodies, we divided nucleosomes affected by H3 depletion into nucleosomes that fell into intergenic or genic regions using annotations from a recent study on the transcribed portion of the yeast genome [13]. For 23 of the 40 data sets shown in Figure 3, the pattern of association with nucleosome behavior was similar between the intergenic and genic regions. That is, factors that were enriched or depleted in a given classification category were enriched or depleted, respectively, in both the intergenic and genic regions. However, the chromatin remodelers Rsc8, Isw2, and Nhp6a were among those that that showed the most striking differences in nucleosome behavior in intergenic regions versus gene bodies (Figure S9 and Table S2). Nhp6a binding was weakly associated with the “occupancy reduced” class in intergenic regions but was strongly associated with the “position altered” and the “occupancy reduced” classes in transcribed regions. This is consistent with a function for Nhp6a in nucleosome incorporation [6] and suggests that areas to which Nhp6a is recruited in transcribed regions may be especially sensitive to a reduction in the available histone pool.

We found that the nucleosomes with reduced occupancy following Nhp6a/b deletion according to [6] were not the same as the nucleosomes classified as “reduced occupancy” in our study after H3 depletion. Only 299 out of the ∼7000 nucleosomes with reduced occupancy following Nhp6a/b deletion in [6] were held in common with the ∼2000 nucleosomes classified as reduced occupancy in our study, suggesting that the mechanisms underlying nucleosome loss in the two experiments are distinct. To examine this more closely, we compared the average Nhp6a binding from [38] to changes in nucleosome occupancy due to H3 depletion (this study) or to changes in nucleosome occupancy due to the absence of Nhp6a/b [6]. While there is a connection between Nhp6a binding and changes in occupancy following H3 depletion in our study (Figure S10A), a connection between Nhp6a binding and nucleosomes lost in in the Nhp6a/b deletion was not apparent (Figure S10C). Similarly, a significant enrichment for Nhp6a binding was found at nucleosomes that belonged to our “reduced occupancy” class (compared to all of the nucleosomes with classified occupancies following H3 depletion; p<1E−15; Figure S10B). However, there was only a weak association between Nhp6a binding and nucleosomes lost in in the Nhp6a/b deletion strain (p = 0.004; Figure S10D).

### DNA sequence has a greater influence on nucleosome occupancy after histone H3 depletion

**Figure 4:**
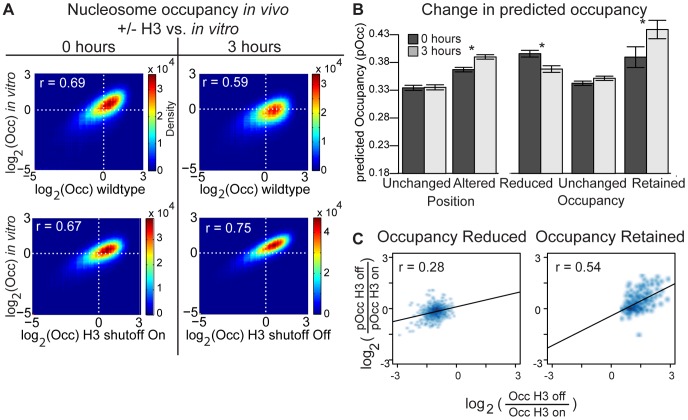
DNA sequence contributes to nucleosome occupancy changes following H3 depletion. (A) *In vivo* nucleosome occupancy versus previously published *in vitro* nucleosome occupancy genome wide [8]. The occupancy values were normalized as in [8], and log_2_ values are plotted. (B) Changes in the DNA sequence-based predicted occupancy (“model score” from [8]) at nucleosomes in the indicated categories at the 0 hour (dark gray) and 3 hour (light gray) nucleosome positions. The error bars indicate the standard error, and pairs marked with an * are significantly different at a p-value<0.001. (C) The degree of nucleosome occupancy reduction or retention following H3 depletion correlates with the DNA-sequence based predictions of change in nucleosome occupancy. Nucleosomes classified as “occupancy reduced” or “occupancy retained” are plotted. Because a position change is required for an alteration in predicted occupancy, nucleosomes classified as “position unchanged” were not included. pOcc = Occupancy prediction from [8]; Occ = occupancy in H3 shutoff strain.

The influence of DNA sequence on nucleosome occupancy has been determined directly by *in vitro* reconstitution of nucleosomes using naked yeast DNA [8], [39]. We compared genome-wide nucleosome occupancy in wildtype and H3 shutoff cells to nucleosome occupancy measured in nucleosome reconstitution experiments. At 0 hours, both wildtype and H3 shutoff cells showed similar correlations to *in vitro* nucleosome reconstitution (r = 0.69 and 0.67, respectively). After 3 hours in dextrose, the wildtype correlation to the *in vitro* data decreased (r = 0.59), but the H3 shutoff strain's correlation increased (r = 0.75; Figure 4A). To confirm that the decreased correlation in dextrose-grown wildtype cells was not specific to our experiment, we compared the correlation between cells grown in galactose and dextrose to the *in vitro* data from an independent strain and data set [8]. A similar decrease in correlation with *in vitro* data was observed between galactose-grown (r = 0.774) and dextrose-grown (r = 0.716) yeast (Figure S11). This suggests that nucleosome occupancy during growth in galactose (as opposed to dextrose) is more similar to the organization observed *in vitro*. More relevant to our study, H3 depletion *in vivo* (which in this case occurs in dextrose) results in a chromatin organization that is much more similar to the *in vitro* configuration than cells with the normal complement of nucleosomes grown in either dextrose or galactose.

We next used a previously published DNA sequence-based model of nucleosome occupancy [8] to determine if the changes in a given nucleosome's position or occupancy after H3 depletion were influenced by the underlying DNA sequence. By definition, occupancy predictions based on DNA-sequence can change only if the nucleosome's position changes. Therefore, we calculated the change in a nucleosome's predicted occupancy based on its position before and after H3 depletion. As expected, the DNA-predicted occupancy value did not change at nucleosomes classified as having unchanged positions because the coordinates for the before and after locations were virtually identical. However, among the “position altered” nucleosomes, there was an average increase in the predicted occupancy at the position following histone depletion relative to the starting position (Figure 4B).

Approximately 25% of the occupancy-altered nucleosomes (reduced or retained) also showed a significant position change following H3 depletion. At these nucleosomes, the average predicted occupancy for the “before” and “after” positions corresponds with the actual observed change in nucleosome occupancy (Figure 4B). To see if this trend extended to all of the nucleosomes with altered occupancy, we examined the change in the actual and predicted occupancies for all of the nucleosomes classified as having altered occupancy and having any degree of shift in position (this group included all position classifications other than “position unchanged”, including “no call”). For both reduced and retained occupancy nucleosomes, the degree of nucleosome reduction or retention *in vivo* correlated with the degree of change in the DNA sequence-predicted nucleosome affinity (Figure 4C). In a recent study that examined nucleosome loss due to Nhp6a/b depletion, the underlying nucleosome-affinity of the DNA also corresponded with changes in nucleosome occupancy [6]. Thus, there is strong evidence that under conditions of limiting histone concentrations, DNA sequence contributes directly to changes in nucleosome occupancy *in vivo*.

### Decreased nucleosome occupancy in promoters following H3 depletion results in increased gene expression

We next revisited the hypothesis that gene expression changes in response to histone depletion are rooted in changes in chromatin organization [1], [3]–[5]. We used RNA-seq to quantify the relative abundance of transcripts from wildtype and H3 shutoff cells after 3 hours in dextrose. Our RNA-seq measurements of relative expression correlated well with previously published microarray experiments from H4-depleted cells, despite differences in histone-depletion methodology and RNA detection methods (r = 0.66 for expression arrays vs. RNA-seq RPMK). We used a BioConductor package, EdgeR, to analyze the RNA-seq data [40]. We detected 2453 de-repressed genes following histone depletion, compared to the 888 that were previously identified [5]. The number of genes with significantly decreased transcript levels following H3 depletion was 753 in this study, compared to the 569 reported previously for H4 depletion (Table S3) [5]. Thus, the increased sensitivity of RNA-seq identified nearly half of yeast genes as having higher expression due to H3 depletion, three times the previous estimate.

**Figure 5:**
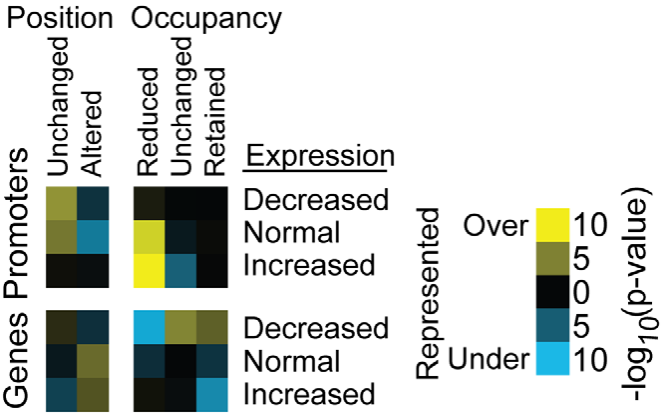
Nucleosomes with reduced occupancy are overrepresented in promoters of genes with increased expression following H3 depletion. The relationship between gene expression changes upon histone depletion (rows) and changes in nucleosome occupancy and position (columns). This relationship is shown for promoters, defined as the untranscribed region upstream of a gene, and genes, defined as the transcribed regions from [13].

We divided the yeast genes into three groups based on expression changes: increased, normal, and decreased. In the promoters of genes with increased or normal expression following H3 depletion, we found an over-representation of “occupancy reduced” nucleosomes (Figure 5 and Figure S12). In contrast, nucleosomes in the promoters of genes with decreased or normal expression were not significantly associated with any class of H3-depletion response. In the gene body, genes with decreased expression tended to harbor “occupancy retained” nucleosomes, while those with increased expression harbored significantly fewer “occupancy retained” nucleosomes than expected (Figure 5 and Figure S12).

## Discussion

The experiments described above support the following main conclusions: (1) Depletion of histone H3 levels causes a defined subset of nucleosomes to alter their position and/or occupancy *in vivo*. (2) Nucleosomes that are preferentially lost tend to be located at promoters, and this, in turn, leads to de-repression of downstream genes. (3) Isw2, an important ATP-dependent chromatin remodeler, is associated with stable nucleosome occupancy but altered position, especially when an adjacent nucleosome is destabilized. Such nucleosomal positioning shifts in the direction of the adjacent loss event are consistent with a nucleosome-centering activity for Isw2 *in vivo*, which to this point has been observed only *in vitro*. (4) Following nucleosome loss, the intrinsic DNA sequence preferences of nucleosomes have a greater influence on occupancy profiles, presumably due to reduced steric hindrance from adjacent nucleosomes. (5) Nhp6a is associated with preferentially lost nucleosomes in gene bodies. Deletion of Nhp6a/b causes a 20–30% reduction in histone abundance and altered transcription of approximately 10% of the yeast genome (fold-change >1.5 and p<0.05) [6]. However, the sets of nucleosomes that we identify as being sensitive to H3 depletion are largely separate from the set identified as being destabilized by the loss of Nhp6a/b. This implies that the nucleosome loss events observed in the two studies may occur by independent mechanisms.

Our approach used MNase-seq to map nucleosome position and relative occupancy before and after H3 depletion. One concern with using MNase to measure nucleosome position and occupancy is that the enzyme's ability to digest DNA can be influenced by the local histone occupancy, with regions of lower histone occupancy being more susceptible to MNase digestion. Following H3 depletion, we observed increased smearing in the MNase-digested DNA fragments but saw no change in the average fragment length or the number of nucleosomes called by our algorithm. Thus, despite the increased sensitivity to MNase, the mononucleosome properties were equivalent to wildtype cells after adjusting the MNase concentration. The strong correlations to biologically relevant annotations provide additional evidence that the position and occupancy comparisons we make based on the data are informative.

We conclude that a combination of DNA-encoded nucleosome preference and chromatin composition regulate nucleosome occupancy and positional stability under conditions of limited histone protein availability. This, in turn, dictates which genes are most susceptible to a loss of regulatory fidelity. Most importantly, our data point to the factors likely to influence nucleosome stability under normal growth conditions, and to the specific genomic locations at which they are likely to act. This information serves as a platform for more detailed investigations into the mechanisms of nucleosome regulation.

## Materials and Methods

### Strains and growth conditions

Wildtype (YEF473A) and H3 shutoff (DCB200.1) cells were maintained on agar plates containing 2% galactose [11]. For experiments, the cells were grown in liquid media with the indicated carbon source. Logarithmically growing cells were diluted to an OD_600_ of 0.0375 in 1.25 L of fresh YPGal media (1% yeast extract, 2% peptone, 2% galactose) and grown for 16 hours. Cells were collected via suction filtration with a 0.2 µm filter and washed with approximately 100 mL YPD (1% yeast extract, 2% peptone, 2% dextrose) before being resuspended in 1.25 L of YPD media. After switching the carbon source, 1 L of cells was immediately transferred to a fresh flask containing formaldehyde for our “0 hour” MNase digested time point (next section). We added 750 mL of fresh YPD to the remaining 250 mL, and the cells were grown at 30°C with shaking for 3 hours prior to collection for the final time point.

### MNase digestion

After collection, the cells were crosslinked with formaldehyde, the cell wall was digested with lyticase, and the DNA was digested with a titration of MNase. The resulting DNA was electrophoresed on an agarose gel, and the mono-nucleosome band was excised for sequencing. See Text S1 for additional details. The raw sequencing data is available at GEO accession number GSE29291 (single-end sequences) and GSE29292 (paired-end sequences).

### RNA isolation and cDNA synthesis

RNA was isolated using the hot acidic phenol method [41] from wildtype and H3 shutoff cells grown as described above and transitioned to dextrose-containing media for 3 hours. RNA was further purified using the RNEasy Mini Kit (Qiagen 74104) to remove trace amounts of phenol. Ribosomal RNA was removed using the RiboMinus system (Invitrogen K155003). The quality of the RNA and the absence of ribosomal RNA were confirmed by gel electrophoresis. We then fragmented 4.5 µg of RNA using Ambion RNA fragmentation reagent (Ambion AM8740) at 70°C for 5 minutes and used the resulting RNA fragments as input for double-strand cDNA synthesis using a double-stranded cDNA synthesis kit (Invitrogen 11917-010) with random priming (Invitrogen 48190-011). The resulting cDNA was then prepared for Illumina sequencing. The raw RNA-seq data is available at GEO accession number GSE29293.

### Illumina GAIIx sequencing

Samples were prepared for either single-end (two H3 shutoff, one wildtype MNase-seq replicates, and three replicates each of H3 shutoff and wildtype RNA data) or paired-end (two H3 shutoff and two wildtype replicates) sequencing using established protocols (Text S1). All libraries were sequenced using an Illumina Genome Analyzer IIx.

### Mapping MNase reads

Reads were aligned to the sacCer1 build of the yeast genome using Bowtie v. 0.12.6 with default settings. Single-end reads were extended to the average fragment size in each experiment, which was calculated based on the distribution of reads on the Watson and Crick strands. Paired-end reads were required to have matching ends within 100–200 bases. Reads aligning to the rDNA locus (chr12 450,000–472,500) were removed. For analysis, all replicates were normalized with regard to sequencing depth by randomly selecting 5 million aligned reads per sample.

### Calling nucleosome position and occupancy

A read center density map was created for each experiment using the center point of each extended (single-end) or paired (paired-end) read. The read-center density map was Gaussian smoothed with a standard deviation (s.d.) of 10 bp and a window of 3 s.d. The overall dyad density was visualized for Figure 1 using Matrix2png [42]. To identify discrete nucleosomes, we repeated the following process: 1) The smoothed read-center density maximum was set as the center of a nucleosome. 2) The size of the region protected by the nucleosome was calculated as the average length of all extended/paired reads that covered the center base. 3) The s.d. of the nucleosome center in bases (the “fuzziness”) of the nucleosome was calculated using the number and locations of read centers that fell within the nucleosome's protected region. 4) The smoothed read-center density for all bases within one protected region of the nucleosome center was set to zero to prevent calling overlapping nucleosomes in successive rounds of nucleosome calling. 5) The nucleosome's occupancy was defined as the number of read centers falling within 50 bp on either side of the nucleosome center. This process was repeated until no additional nucleosomes could be called.

### Classifying nucleosome behavior

Nucleosome positions at 0 and 3 hours in each replicate were required to overlap by 30 bp, and the coverage in each case was required to be greater than 5 reads. The nucleosome center positions at 0 and 3 hours were subjected to a t-test, and a p-value was calculated. The occupancy was compared using the binomial distribution test on the number of read centers in the 100-bp window centered on the nucleosomes' respective centers. In both cases, the resulting p-values were Bonferroni corrected based on the total number of nucleosomes compared in that replicate. Nucleosomes for each replicate were classified as having either unchanged (p>0.2) or altered (p<0.01) position and reduced (p<0.01), unchanged (p>0.2 and <0.8), or retained (p>0.99) occupancy relative to the 0 hour position and occupancy. Nucleosomes with p-values between 0.01 and 0.2 or 0.8 and 0.99 were left unclassified (“no call”) and were not used in downstream analyses. Nucleosome classification was compared between replicates, and only nucleosomes that were similarly classified in two or more replicates were used for further analyses. In addition, occupancy categories were restricted to nucleosomes that were not oppositely classified in any replicates (i.e., no nucleosomes were considered that were classified as occupancy reduced and occupancy retained in different replicates). Nucleosomes exhibiting behavior that was dependent on histone H3 depletion were identified by comparing the wildtype and H3 shutoff strains and removing any nucleosomes that were similarly classified in the wildtype experiments.

### Enrichment/depletion analysis

The majority of data sets used for comparative analysis were also generated from cells grown in glucose at an OD600 of ∼1. If the growth condition in the study was substantially different, it is noted in parentheses. We compared the nucleosome classes to available genome-wide data sets for the following marks that were downloaded from ChromatinDB (www.bioinformatics2.wsu.edu/cgi-bin/ChromatinDB/cgi/downloader_select.pl) as bulk histone occupancy normalized data: H2AK7ac, H2BK11ac, H2BK16ac, H3K9ac, H3K14ac, H3K18ac, H3K23ac, H3K27ac, H4K8ac, H4K12ac and H4K16ac [43]; H3 N-terminal ac and H4 N-terminal acetylation [44]; H3K36me2 [45]; H2A.zK14ac [46]; H3K56ac [34], [47]; and H3 and H4 occupancy [48]. We also used H3K36ac [49] and H3K4me3 [50] data normalized to H3 occupancy from [48]. H3K4me2, H3K4me4, and H3R2me2a H3-normalized data is from [51]. H3 turnover data is from [20] (grown in raffinose and galactose). Sir2 data is from [52]. Tup1 data is from [21] (OD_600_∼0.6–0.8). Rpo21, Bdf1, Rap1, Reb1, Vps72, and Srm1 data is from [22]. Rsc8 data is from [53]. Isw2K215R data is from [32] (OD_600_∼0.7). Nhp6a data is from [38]. Rpd3 data is from [54]. High resolution H2A.z data is from [55]. For Figure 3 and Figure S7, the average enrichment of the comparison data for nucleosomes in each class was calculated. To calculate significance, the average enrichment for an identical number of nucleosomes selected from the entire set of H3 depletion-dependent nucleosomes was calculated 100 times, and the standard deviation of the random average was used to assign a z-score for the actual value. The data is reported as the −log_10_ of the p-value of the z-score (raw z-scores are available in Table S2 and Table S3).

### Comparison to Nhp6a/b depletion

H3-depletion affected histones identified in this study were compared to nucleosomes identified in a previous study in which Nhp6a and Nhp6b were deleted in a yeast strain [6]. To compare changes in occupancy between the two experiment sets, we used the log_2_ ratio of the Nhp6a/b deletion strain's score to the wildtype score for the Celona et al. data [6] and the previously described classification of H3-depletion dependent occupancy.

### RNA processing

RNA-seq reads from three biological replicates each of H3 shutoff and wildtype cells were mapped to the *S. cerevisiae* sacCer1 genome using Bowtie (v. 0.12.6.0) and TopHat (v. 1.1.0) with a maximum intron size of 1 kb. Samtools (v. 0.1.8.0) was used to determine read pileups at each base in the genome. The total coverage in bases reported as transcribed [13] was divided by the read length (28 bp) and was used as the coverage for determining differential gene expression using EdgeR [40]. Genes with a reported p-value<0.001 were considered to be differentially expressed.

### Histone depletion–dependent changes versus expression changes

The number of nucleosomes in each class that overlapped with each gene and upstream, non-transcribed region was determined. To calculate significance, a number of nucleosomes equal to those in the class were randomly chosen from all classified nucleosomes, and this subset was tested for overlap with the region. This was repeated 100 times, and the −log_10_ p-value of the z-score is reported.

## Supporting Information

Figure S1 Transition to dextrose silences *GAL-H3* transcription. (A) Wildtype (YEF473A; green) and H3 shutoff (DCB200.1; blue) strains grow at similar rates in media containing galactose. Values reported are the cell count relative to the 0 hour time point for each strain. (B) Although the H3 shutoff cells (red) undergo DNA synthesis, they arrest in large-budded phase prior to a complete cell division in dextrose-containing media while the wildtype cells (brown) continue to divide. All counts are reported relative to the 0 hour time point for each strain. See [11] for additional details. (C) After 3 hours in dextrose, H3 (*HHT2*) transcription is nearly silent compared to wildtype cells. Note that the transcription of the other histone proteins (H4: *HHF1* and *HHF2*; H2A: *HTA1* and *HTA2*; and H2B: *HTB1* and *HTB2*) is unaffected. Transcription of G6PDH (*ZWF1* in yeast) is not repressed in the H3 shutoff strain following the shift to dextrose. All bars indicate the average reads per thousand mapped tags (RPKM) from three RNA-seq experiments. The error bars indicate the standard error. (D) H3 protein levels were reduced to ∼50% of the original level after 3 hours in dextrose. A representative digital western blot image and the quantification of three blot replicates (bar plot) are shown. G6PDH was uniform over the time course in the H3 shutoff strain (normalized to the 0 hour time point). The reported H3 levels following nucleosome depletion are normalized to G6PDH at that time point prior to normalization against the 0 hour H3 level. The average of three replicates is reported along with the standard error.
(TIF)

Figure S2 Experimental design for measuring effect of H3 depletion. (A) Cultures of either wildtype or H3 shutoff cells were initially grown to an OD_600_ between 0.8 and 1 in galactose media. (B) Cells were then isolated by filtration, rinsed with dextrose media, and resuspended at the original density in fresh dextrose media. A sample was removed for MNase digestion. (C) Cells were diluted 1∶4 in dextrose media and incubated at 30°C for 3 hours before being digested with MNase. (D, E) 0 hour MNase digest gels for wildtype and H3 shutoff cells. (F, G) MNase digest gels for wildtype and H3 shutoff cells after 3 hours in dextrose. No clear tri-nucleosome band is seen for H3 shutoff cells, and there is increased background smearing (Text S1, p<0.05). (H, I) Correlation coefficient for all wildtype experiments at 0 and 3 hours (A: single-end sequencing (red); B, C: paired-end sequencing (blue)). (J, K) Correlation coefficients for all of the H3 shutoff experiments at 0 and 3 hours [A, B: single end sequencing (red); C, D: paired end sequencing (blue)].
(TIF)

Figure S3 Stringent identification of H3-depletion responsive nucleosomes. Nucleosomes were first classified in each replicate in H3 shutoff (“H3 Depletion Replicates”, top) and wildtype cells (“Wildtype Replicates”, bottom). Nucleosomes that behaved consistently in two or more replicates and met other quality-control measures (see Materials and Methods) were selected as behaving consistently (see “H3 Depletion Consistent” and “Wildtype Consistent” rows). Nucleosomes in the “H3 Depletion Consistent” classes that were not found in the “Wildtype Consistent” classification were selected as “H3 Depletion Dependent” nucleosomes and used for further analyses.
(TIF)

Figure S4 Nucleosome rearrangements upstream of *PHO5* are linked to increased *PHO5* expression. (A) Independent MNase-seq experiments [8] in YPGal (black) and YPD (pink) at the *PHO5*/*PBY1* divergent promoter. (B) Nucleosome positions as called in [56] for comparison. (C) Southern blot probes used in [1], in which nucleosome occupancy upstream of the *PHO5* promoter was found to be disrupted. (D–G). Normalized nucleosome occupancy for each of our four H3 shutoff replicates in YPGal (blue, green, gray, and purple, respectively) and YPD (red, orange, bright green, pink, respectively) with the nucleosome position calls made by our algorithm shown below. Our algorithm identified either two (Reps A and C) or three (reps B and D) nucleosomes between DNase hypersensitivity sites 1 and 2 (HS1 and HS2, shown in A). Numbering nucleosomes backwards from the *PHO5* gene, the −1 and −2 nucleosomes that flank HS1 were relatively unaffected by the histone shutoff, but nucleosomes −3 through −5, which were the nucleosomes measured by the probes in Han et al. [1], were rearranged following H3 depletion.
(TIF)

Figure S5 H3 depletion alters chromatin structure around the *IDP2* transcription start site. The *IDP2* gene experienced an H3-depletion dependent increase in transcription. We chose this locus as an example to illustrate our classification process. (A) Normalized wildtype nucleosome occupancy profiles generated in YPGal (black) and YPD (pink) from [8], a completely independent set of MNase digestions. (B) Normalized nucleosome occupancy profiles for a single replicate of wildtype cells (replicate C) grown in YPGal (green) and YPD (orange). (C) Normalized nucleosome occupancy profiles for a single replicate of H3 shutoff cells (replicate C) grown in YPGal (blue) or YPD (red). (D) Nucleosomes called from the wildtype and H3 shutoff data in YPGal (green and blue) and YPD (orange and red) data. The position and occupancy of the 3 nucleosomes immediately surrounding the transcription start site (nucleosomes 3–5) are disrupted. The ovals indicate the precise width of the nucleosome-protected region as called using the paired-end data. The intensity of the color (YPGal dark and YPD light, both dark, YPGal light and YPD dark, or both light) indicates the nucleosome occupancy call (reduced, unchanged, retained, or no call, respectively), while the outline indicates the positional call (both solid, unchanged; YPGal solid and YPD dashed, altered; no outline, no call). (E) The positional change (“Center diff”), position standard error (“Pos s.e.”) and occupancy counts (“YPGal Occ” and “YPD Occ”) for each nucleosome represented in (D) are given, along with the Bonferroni multiple-testing corrected p-values. The number of nucleosomes in the replicate is given below as the multiple-testing correction factor. Significant p-values are shown in red. P-values that fall into the “no call” category are gray. For position, t-test p-values<0.01 were classified as altered, while p-values>0.2 were classified as unchanged. For occupancy, a two-tailed binomial distribution test was used. Occupancy p-values<0.01 were classified as reduced, p-values between 0.2 and 0.8 (64,853.8 for wildtype or 68,567.8 for H3 shutoff after correction) were unchanged, and p-values>0.99 (64,853.99 for wildtype or 68,567.99 for H3 shutoff after correction) were classified as preferentially retained.
(TIF)

Figure S6 Enrichment for factors in H3-depletion effect categories. The average value of previously published genome-wide data sets and genome annotations vs. expected (Z-score) for each of the nucleosome change categories shown in Figure 3 (Materials and Methods). (A) Genome annotations, (B) histone modifications, (C) transcription-associated proteins, (D) chromatin remodelers and (E) histones. The number in parenthesis indicates the resolution of the detection platform in the data set used for comparison.
(TIF)

Figure S7 H3 turnover is associated with nucleosome loss. (A) Comparison of nucleosome's H3-depletion dependent occupancy change vs. the H3 turnover Z-score from [20]. Nucleosomes classified as “occupancy reduced”, which were found to be enriched for H3 turnover, are highlighted in red. (B) Distribution of the H3 turnover Z-score for each category. Consistent with the results in Figure 3, the “occupancy reduced” and “occupancy unchanged” categories were found to be statistically different than the value for all H3 depletion affected nucleosomes. * p-value<1×10^−8^; ** p-value<1×10^−15^.
(TIF)

Figure S8 Isw2-bound altered position nucleosomes are centered on available DNA. (A) Schematic representation of the hypothesized nucleosome-centering function of Isw2 (blue oval) following histone (yellow circle) depletion. (B, C) Nucleosomes that fell within regions identified as bound by Isw2K215R in [32] that showed (B) altered position following H3 depletion were aligned based on their 0 hour position (y-axis at bottom) and were oriented according to the direction of the position change following H3 depletion. Each row was aligned so that the direction of change is to the right. The fold change in nucleosome occupancy 600 bp upstream and downstream is indicated in the heatmap. Note the blue vertical stripe to the left and the yellow stripe to the right, indicating the shift of the nucleosome position. Shown below with matched coordinates is the average nucleosome occupancy at 0 hours (blue) and 3 hours (red). (C) Same as (B) but for nucleosomes bound by Isw2K215R with unchanged position (D) Box plots indicating the upstream (Direction U) and downstream (Direction D) distance to the nearest nucleosome for position-altered nucleosomes that are either bound by Isw2K215R (blue) or not (green). The darker shade indicates the distance prior to histone depletion, while the lighter shades indicate the distance following H3 depletion. Notches indicate 95% confidence intervals for the data.
(TIF)

Figure S9 Context-dependent associations of chromatin marks and remodelers. (A) Similar enrichment patterns are seen for transcription-associated factors regardless of if the nucleosome falls in an intergenic or genic region. (B) Chromatin remodelers show altered associations with H3 depletion effects in intergenic and coding regions. (C) Histone protein enrichment levels are similar regardless of whether the nucleosome falls in an intergenic or genic region. (D) The histone modification status affects outcome primarily at nucleosomes that occur in genic regions. For all sections, the number in parentheses indicates the technical resolution of the data set used for comparison.
(TIF)

Figure S10 Nhp6a enrichment is associated with nucleosome loss in H3-depleted cells. We compared changes in nucleosome occupancy as the result of H3 depletion (this study) or deletion of Nhp6a/b [6] to previously measured levels of Nhp6a binding [38]. (A) “Occupancy reduced” nucleosomes (red) show a higher level of Nhp6a binding than the other occupancy classified nucleosomes. (B) Nhp6a-binding levels by H3 depletion occupancy categories shows increased and decreased levels of Nhp6a at “occupancy reduced” and “occupancy unchanged” nucleosomes. Notches indicate 95% confidence levels around the median. (C) As in A except using nucleosome scores from a study in which Nhp6a/b were deleted [6] and nucleosomes with a log_2_ ratio of WT to Nhp6a/b deleted cells <−2 are highlighted in orange. (D) As in B with classifications of “decreased” (log_2_ ratio<−2), “no change” (log_2_ ratio >−0.5 and <0.5), and “increased” (log_2_ ratio >2). * p-value <0.01; ** p-value<1×10^−5^.
(TIF)

Figure S11 Nucleosome occupancy in galactose-grown cells is more similar to *in vitro* reconstituted nucleosome occupancy than dextrose-grown cells. (A) *In vivo* nucleosome occupancy from yeast grown in galactose versus previously published *in vitro* nucleosome occupancy genome wide [8]. The occupancy values were normalized as in [8]; log_2_ values are plotted. (B) Same as (A), but for yeast grown in dextrose.
(TIF)

Figure S12 Association of H3-depletion effect categories with the transcriptional response. The relationship between gene expression changes upon histone depletion (rows) and changes in nucleosome occupancy and position (columns) as in Figure 5, except that the colors indicate the Z-score for enrichment rather than the log_10_ of the p-value. This relationship is shown for promoters, defined as the untranscribed region upstream of a gene, and genes, defined as the transcribed regions from [13].
(TIF)

Table S1 Enrichment of histone modifications and chromatin associated proteins by nucleosome class.
(XLS)

Table S2 Enrichment of histone modifications and chromatin associated proteins by nucleosome class split by genomic annotation.
(XLS)

Table S3 Expression changes by gene following H3 depletion.
(XLS)

Text S1 Additional details regarding the western blots, MNase digestions, calculations regarding MNase susceptibility, and details on single-end and paired-end library preparations.
(DOCX)
