# Supplementary figures and images for: In Vivo Effects of Histone H3 Depletion on Nucleosome Occupancy and Position in Saccharomyces cerevisiae
Source: PLoS Genet. 2012 Jun 21;8(6):e1002771. doi: 10.1371/journal.pgen.1002771 (PMC3380831; doi:10.1371/journal.pgen.1002771)

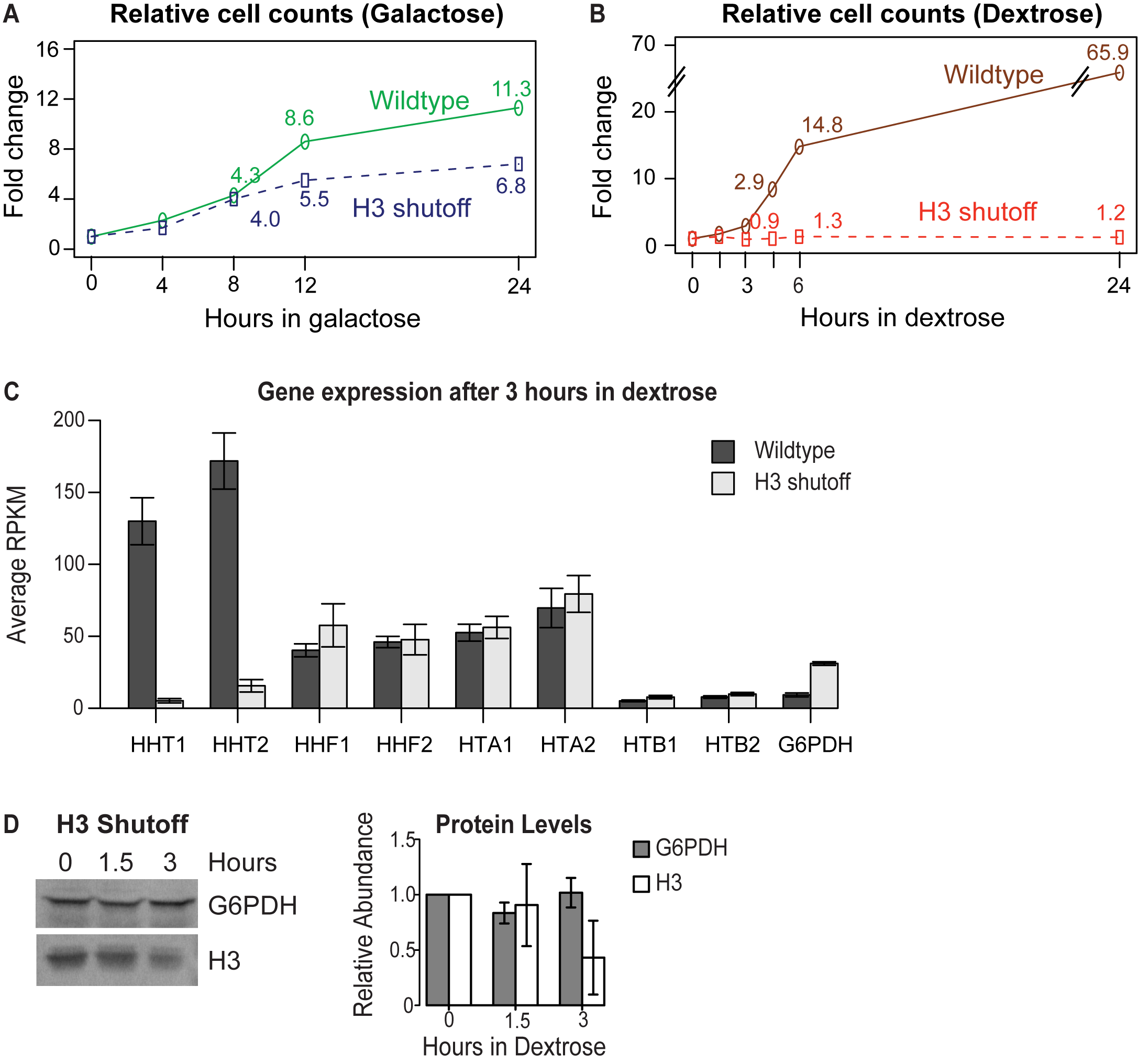

Supplement: Figure S1 — Transition to dextrose silences GAL-H3 transcription. (A) Wildtype (YEF473A; green) and H3 shutoff (DCB200.1; blue) strains grow at similar rates in media containing galactose. Values reported are the cell count relative to the 0 hour time point for each strain. (B) Although the H3 shutoff cells (red) undergo DNA synthesis, they arrest in large-budded phase prior to a complete cell division in dextrose-containing media while the wildtype cells (brown) continue to divide. All counts are reported relative to the 0 hour time point for each strain. See [11] for additional details. (C) After 3 hours in dextrose, H3 (HHT2) transcription is nearly silent compared to wildtype cells. Note that the transcription of the other histone proteins (H4: HHF1 and HHF2; H2A: HTA1 and HTA2; and H2B: HTB1 and HTB2) is unaffected. Transcription of G6PDH (ZWF1 in yeast) is not repressed in the H3 shutoff strain following the shift to dextrose. All bars indicate the average reads per thousand mapped tags (RPKM) from three RNA-seq experiments. The error bars indicate the standard error. (D) H3 protein levels were reduced to ∼50% of the original level after 3 hours in dextrose. A representative digital western blot image and the quantification of three blot replicates (bar plot) are shown. G6PDH was uniform over the time course in the H3 shutoff strain (normalized to the 0 hour time point). The reported H3 levels following nucleosome depletion are normalized to G6PDH at that time point prior to normalization against the 0 hour H3 level. The average of three replicates is reported along with the standard error. (TIF) [file pgen.1002771.s001.tif]

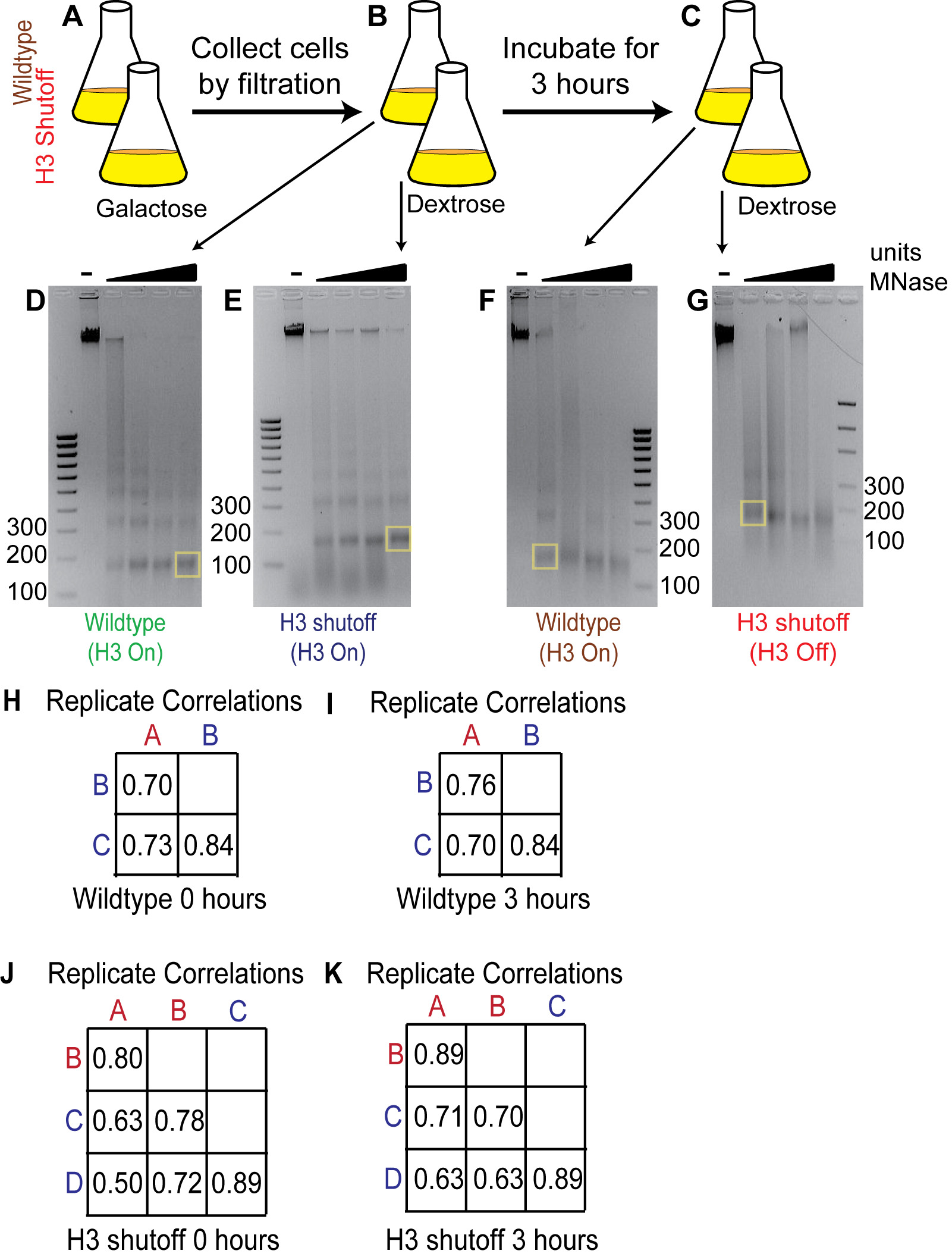

Supplement: Figure S2 — Experimental design for measuring effect of H3 depletion. (A) Cultures of either wildtype or H3 shutoff cells were initially grown to an OD600 between 0.8 and 1 in galactose media. (B) Cells were then isolated by filtration, rinsed with dextrose media, and resuspended at the original density in fresh dextrose media. A sample was removed for MNase digestion. (C) Cells were diluted 1∶4 in dextrose media and incubated at 30°C for 3 hours before being digested with MNase. (D, E) 0 hour MNase digest gels for wildtype and H3 shutoff cells. (F, G) MNase digest gels for wildtype and H3 shutoff cells after 3 hours in dextrose. No clear tri-nucleosome band is seen for H3 shutoff cells, and there is increased background smearing (Text S1, p<0.05). (H, I) Correlation coefficient for all wildtype experiments at 0 and 3 hours (A: single-end sequencing (red); B, C: paired-end sequencing (blue)). (J, K) Correlation coefficients for all of the H3 shutoff experiments at 0 and 3 hours [A, B: single end sequencing (red); C, D: paired end sequencing (blue)]. (TIF) [file pgen.1002771.s002.tif]

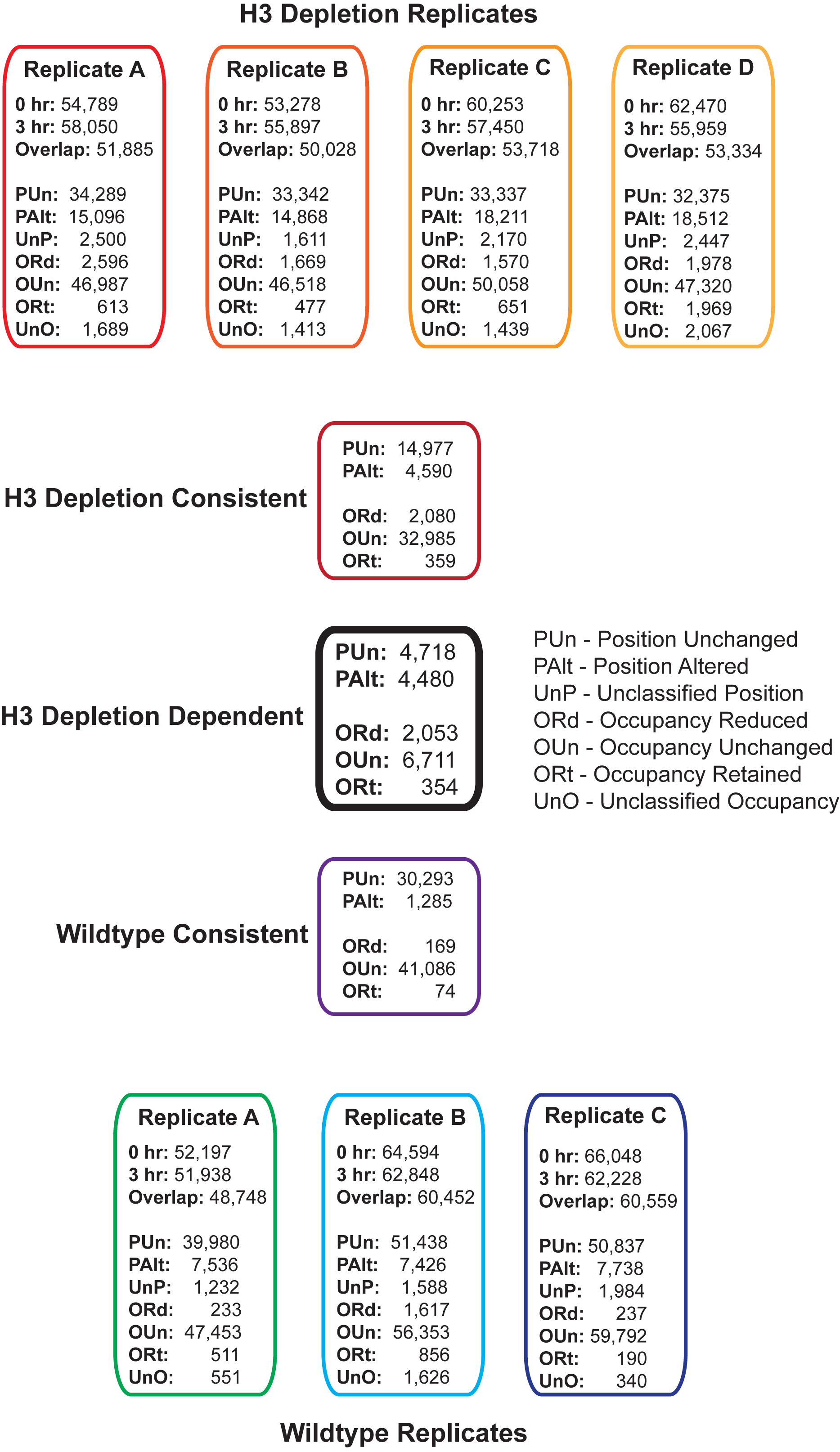

Supplement: Figure S3 — Stringent identification of H3-depletion responsive nucleosomes. Nucleosomes were first classified in each replicate in H3 shutoff (“H3 Depletion Replicates”, top) and wildtype cells (“Wildtype Replicates”, bottom). Nucleosomes that behaved consistently in two or more replicates and met other quality-control measures (see Materials and Methods) were selected as behaving consistently (see “H3 Depletion Consistent” and “Wildtype Consistent” rows). Nucleosomes in the “H3 Depletion Consistent” classes that were not found in the “Wildtype Consistent” classification were selected as “H3 Depletion Dependent” nucleosomes and used for further analyses. (TIF) [file pgen.1002771.s003.tif]

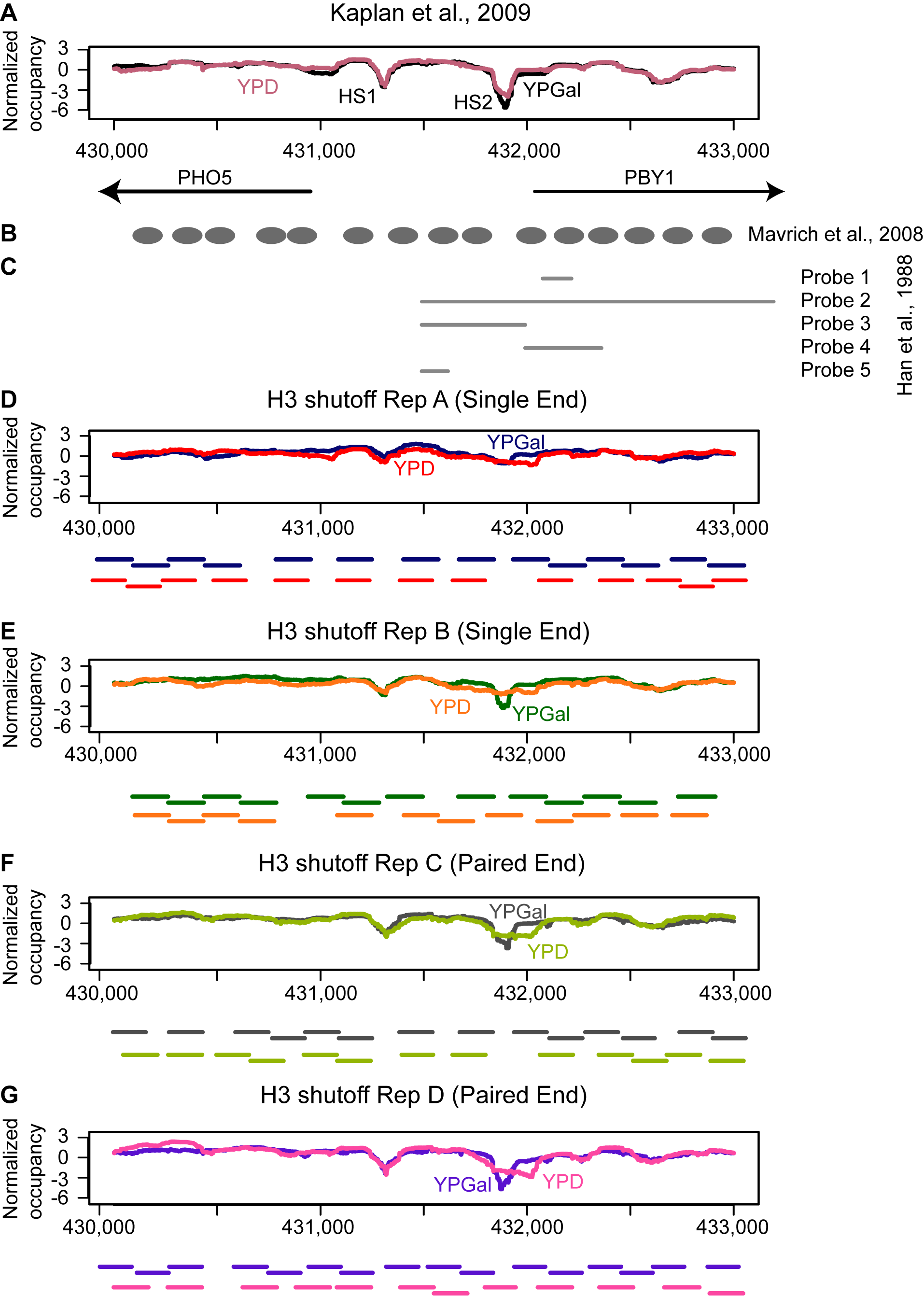

Supplement: Figure S4 — Nucleosome rearrangements upstream of PHO5 are linked to increased PHO5 expression. (A) Independent MNase-seq experiments [8] in YPGal (black) and YPD (pink) at the PHO5/PBY1 divergent promoter. (B) Nucleosome positions as called in [56] for comparison. (C) Southern blot probes used in [1], in which nucleosome occupancy upstream of the PHO5 promoter was found to be disrupted. (D–G). Normalized nucleosome occupancy for each of our four H3 shutoff replicates in YPGal (blue, green, gray, and purple, respectively) and YPD (red, orange, bright green, pink, respectively) with the nucleosome position calls made by our algorithm shown below. Our algorithm identified either two (Reps A and C) or three (reps B and D) nucleosomes between DNase hypersensitivity sites 1 and 2 (HS1 and HS2, shown in A). Numbering nucleosomes backwards from the PHO5 gene, the −1 and −2 nucleosomes that flank HS1 were relatively unaffected by the histone shutoff, but nucleosomes −3 through −5, which were the nucleosomes measured by the probes in Han et al. [1], were rearranged following H3 depletion. (TIF) [file pgen.1002771.s004.tif]

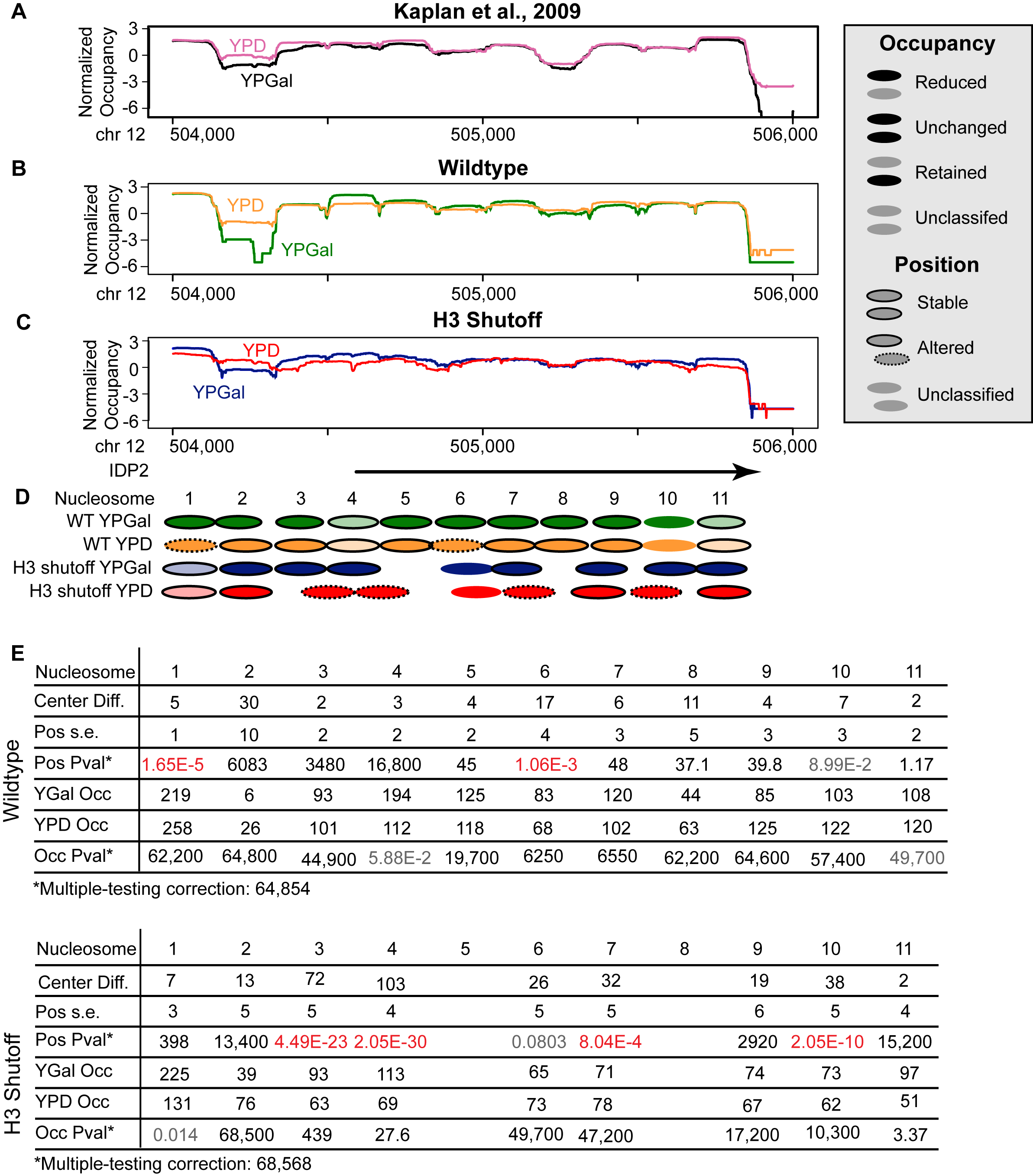

Supplement: Figure S5 — H3 depletion alters chromatin structure around the IDP2 transcription start site. The IDP2 gene experienced an H3-depletion dependent increase in transcription. We chose this locus as an example to illustrate our classification process. (A) Normalized wildtype nucleosome occupancy profiles generated in YPGal (black) and YPD (pink) from [8], a completely independent set of MNase digestions. (B) Normalized nucleosome occupancy profiles for a single replicate of wildtype cells (replicate C) grown in YPGal (green) and YPD (orange). (C) Normalized nucleosome occupancy profiles for a single replicate of H3 shutoff cells (replicate C) grown in YPGal (blue) or YPD (red). (D) Nucleosomes called from the wildtype and H3 shutoff data in YPGal (green and blue) and YPD (orange and red) data. The position and occupancy of the 3 nucleosomes immediately surrounding the transcription start site (nucleosomes 3–5) are disrupted. The ovals indicate the precise width of the nucleosome-protected region as called using the paired-end data. The intensity of the color (YPGal dark and YPD light, both dark, YPGal light and YPD dark, or both light) indicates the nucleosome occupancy call (reduced, unchanged, retained, or no call, respectively), while the outline indicates the positional call (both solid, unchanged; YPGal solid and YPD dashed, altered; no outline, no call). (E) The positional change (“Center diff”), position standard error (“Pos s.e.”) and occupancy counts (“YPGal Occ” and “YPD Occ”) for each nucleosome represented in (D) are given, along with the Bonferroni multiple-testing corrected p-values. The number of nucleosomes in the replicate is given below as the multiple-testing correction factor. Significant p-values are shown in red. P-values that fall into the “no call” category are gray. For position, t-test p-values<0.01 were classified as altered, while p-values>0.2 were classified as unchanged. For occupancy, a two-tailed binomial distribution test was used. Occupancy p-valu [file pgen.1002771.s005.tif]

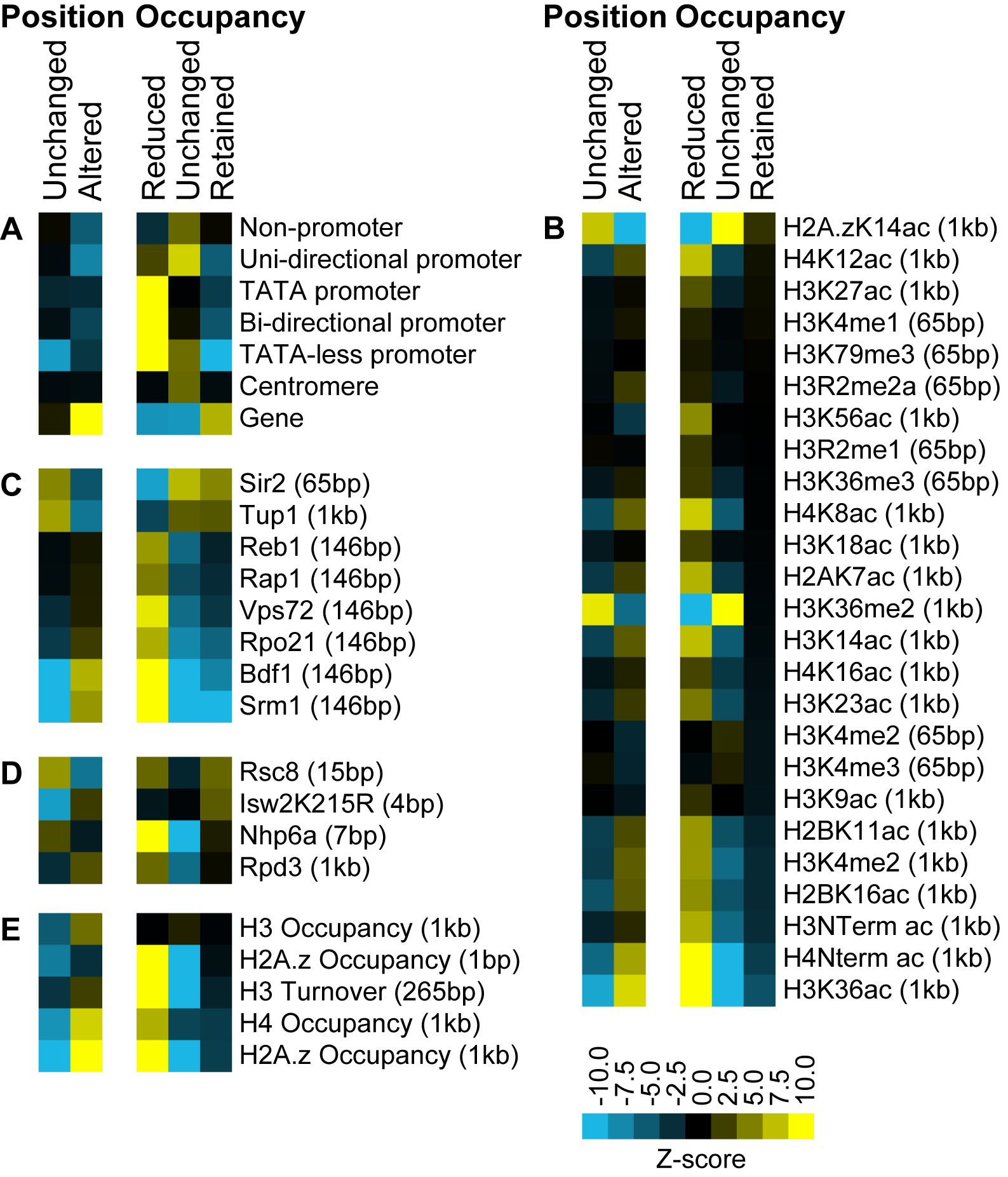

Supplement: Figure S6 — Enrichment for factors in H3-depletion effect categories. The average value of previously published genome-wide data sets and genome annotations vs. expected (Z-score) for each of the nucleosome change categories shown in Figure 3 (Materials and Methods). (A) Genome annotations, (B) histone modifications, (C) transcription-associated proteins, (D) chromatin remodelers and (E) histones. The number in parenthesis indicates the resolution of the detection platform in the data set used for comparison. (TIF) [file pgen.1002771.s006.tif]

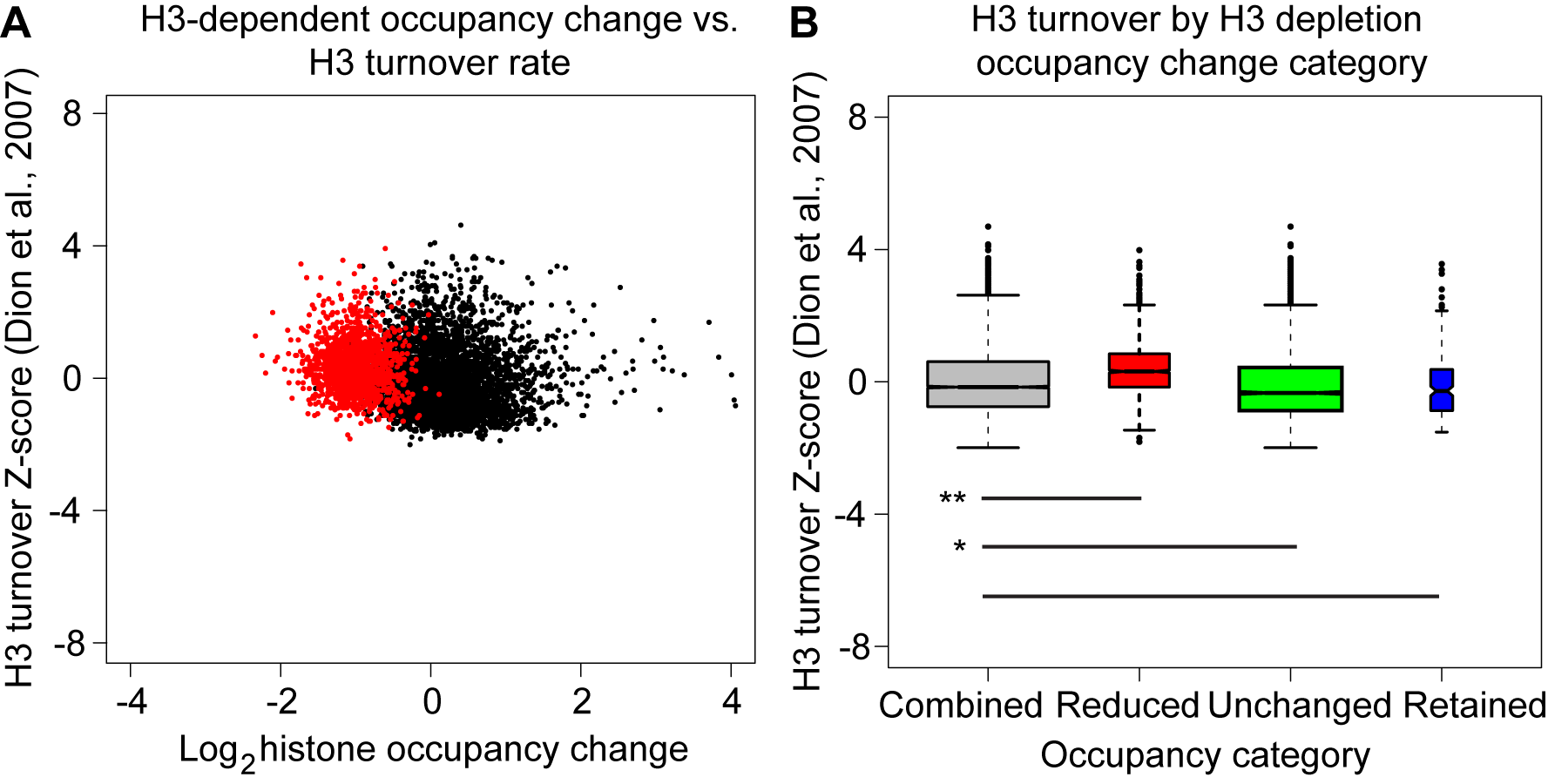

Supplement: Figure S7 — H3 turnover is associated with nucleosome loss. (A) Comparison of nucleosome's H3-depletion dependent occupancy change vs. the H3 turnover Z-score from [20]. Nucleosomes classified as “occupancy reduced”, which were found to be enriched for H3 turnover, are highlighted in red. (B) Distribution of the H3 turnover Z-score for each category. Consistent with the results in Figure 3, the “occupancy reduced” and “occupancy unchanged” categories were found to be statistically different than the value for all H3 depletion affected nucleosomes. * p-value<1×10^−8^; ** p-value<1×10^−15^. (TIF) [file pgen.1002771.s007.tif]

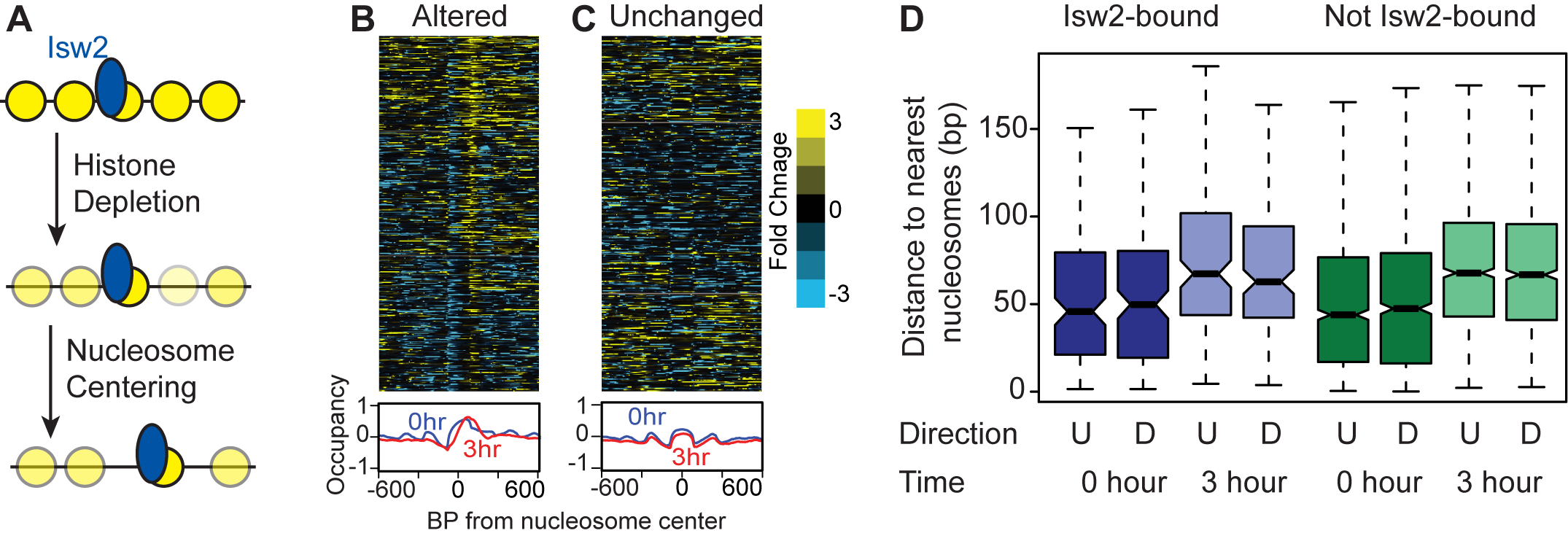

Supplement: Figure S8 — Isw2-bound altered position nucleosomes are centered on available DNA. (A) Schematic representation of the hypothesized nucleosome-centering function of Isw2 (blue oval) following histone (yellow circle) depletion. (B, C) Nucleosomes that fell within regions identified as bound by Isw2K215R in [32] that showed (B) altered position following H3 depletion were aligned based on their 0 hour position (y-axis at bottom) and were oriented according to the direction of the position change following H3 depletion. Each row was aligned so that the direction of change is to the right. The fold change in nucleosome occupancy 600 bp upstream and downstream is indicated in the heatmap. Note the blue vertical stripe to the left and the yellow stripe to the right, indicating the shift of the nucleosome position. Shown below with matched coordinates is the average nucleosome occupancy at 0 hours (blue) and 3 hours (red). (C) Same as (B) but for nucleosomes bound by Isw2K215R with unchanged position (D) Box plots indicating the upstream (Direction U) and downstream (Direction D) distance to the nearest nucleosome for position-altered nucleosomes that are either bound by Isw2K215R (blue) or not (green). The darker shade indicates the distance prior to histone depletion, while the lighter shades indicate the distance following H3 depletion. Notches indicate 95% confidence intervals for the data. (TIF) [file pgen.1002771.s008.tif]

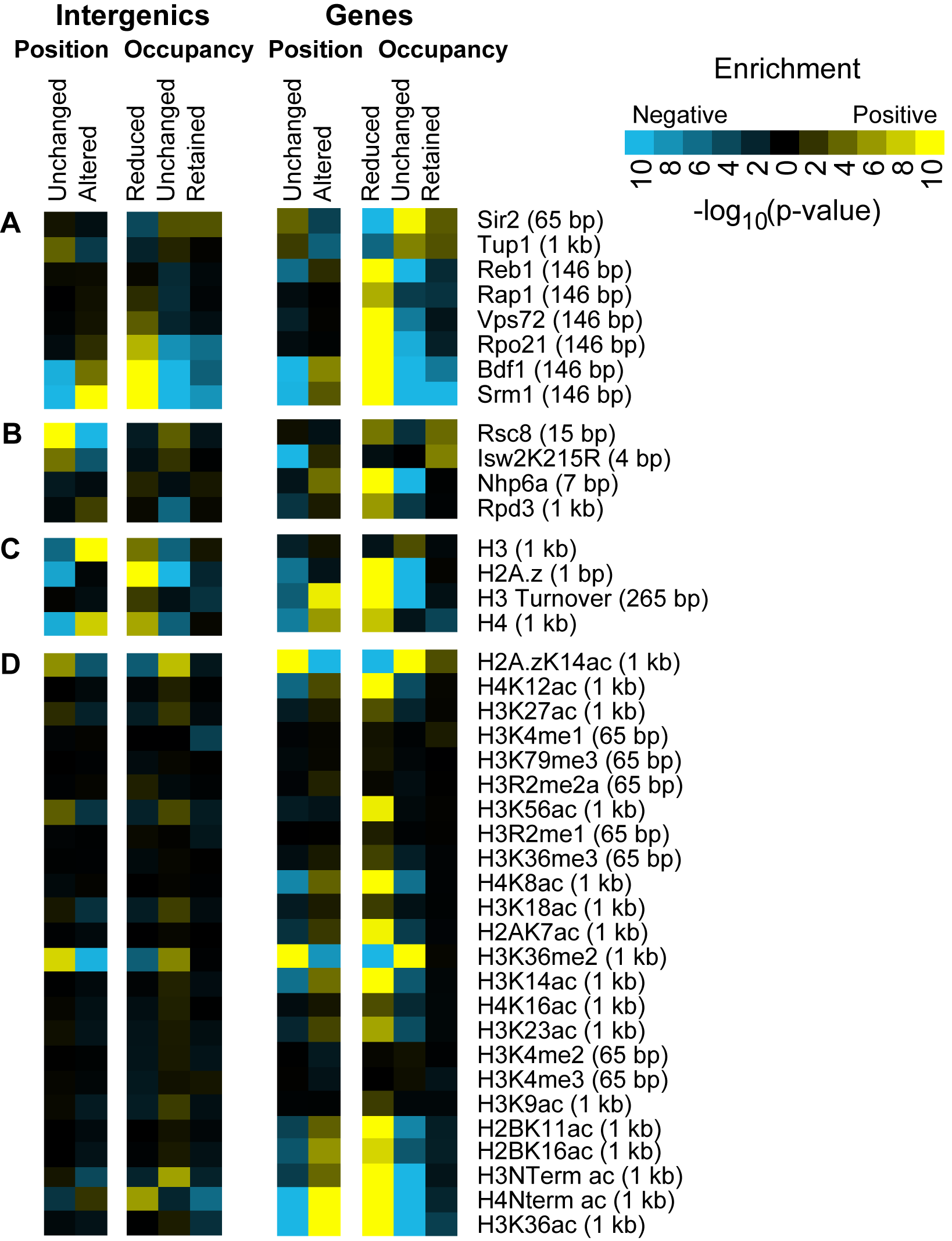

Supplement: Figure S9 — Context-dependent associations of chromatin marks and remodelers. (A) Similar enrichment patterns are seen for transcription-associated factors regardless of if the nucleosome falls in an intergenic or genic region. (B) Chromatin remodelers show altered associations with H3 depletion effects in intergenic and coding regions. (C) Histone protein enrichment levels are similar regardless of whether the nucleosome falls in an intergenic or genic region. (D) The histone modification status affects outcome primarily at nucleosomes that occur in genic regions. For all sections, the number in parentheses indicates the technical resolution of the data set used for comparison. (TIF) [file pgen.1002771.s009.tif]

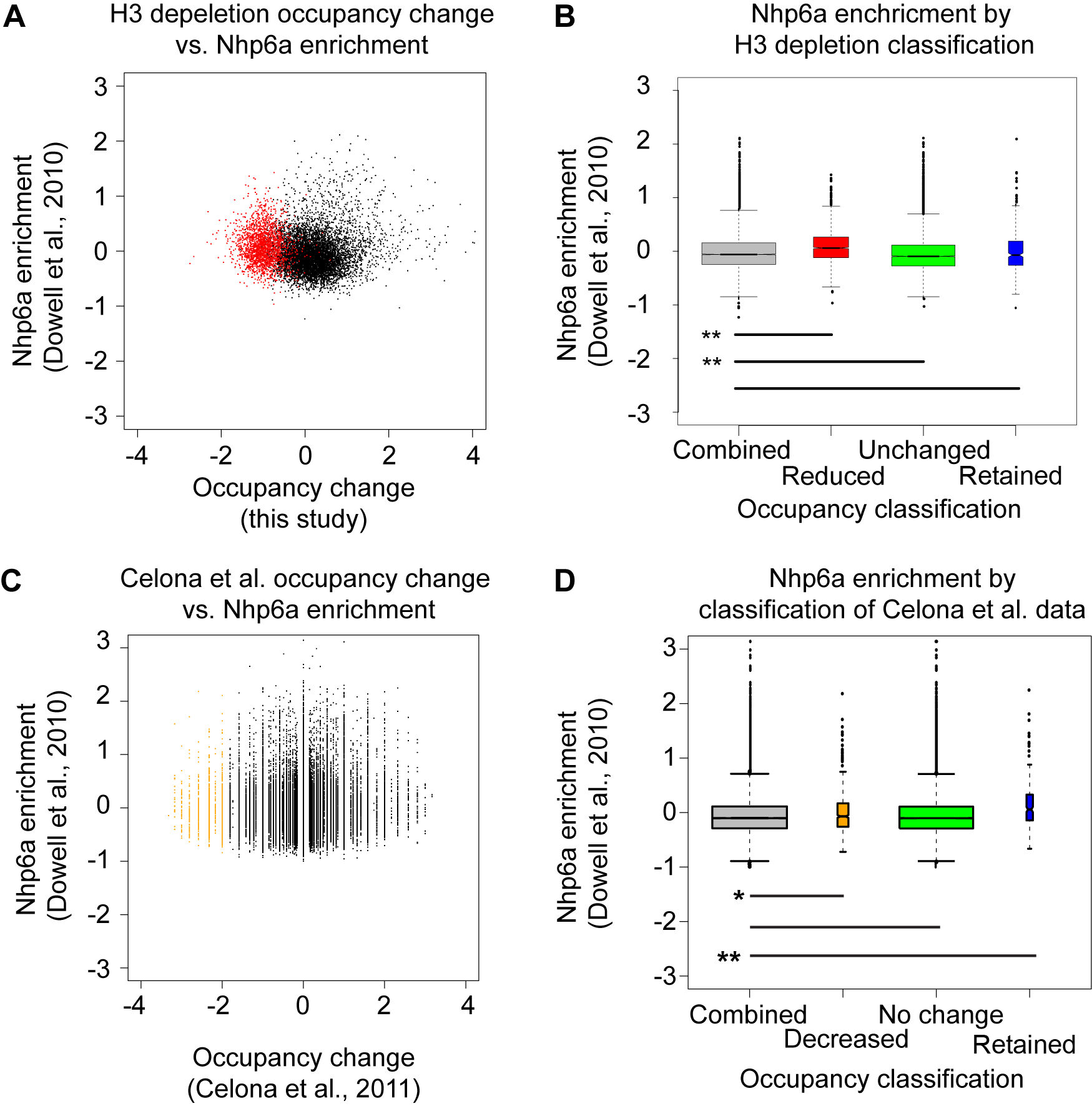

Supplement: Figure S10 — Nhp6a enrichment is associated with nucleosome loss in H3-depleted cells. We compared changes in nucleosome occupancy as the result of H3 depletion (this study) or deletion of Nhp6a/b [6] to previously measured levels of Nhp6a binding [38]. (A) “Occupancy reduced” nucleosomes (red) show a higher level of Nhp6a binding than the other occupancy classified nucleosomes. (B) Nhp6a-binding levels by H3 depletion occupancy categories shows increased and decreased levels of Nhp6a at “occupancy reduced” and “occupancy unchanged” nucleosomes. Notches indicate 95% confidence levels around the median. (C) As in A except using nucleosome scores from a study in which Nhp6a/b were deleted [6] and nucleosomes with a log2 ratio of WT to Nhp6a/b deleted cells <−2 are highlighted in orange. (D) As in B with classifications of “decreased” (log2 ratio<−2), “no change” (log2 ratio >−0.5 and <0.5), and “increased” (log2 ratio >2). * p-value <0.01; ** p-value<1×10^−5^. (TIF) [file pgen.1002771.s010.tif]

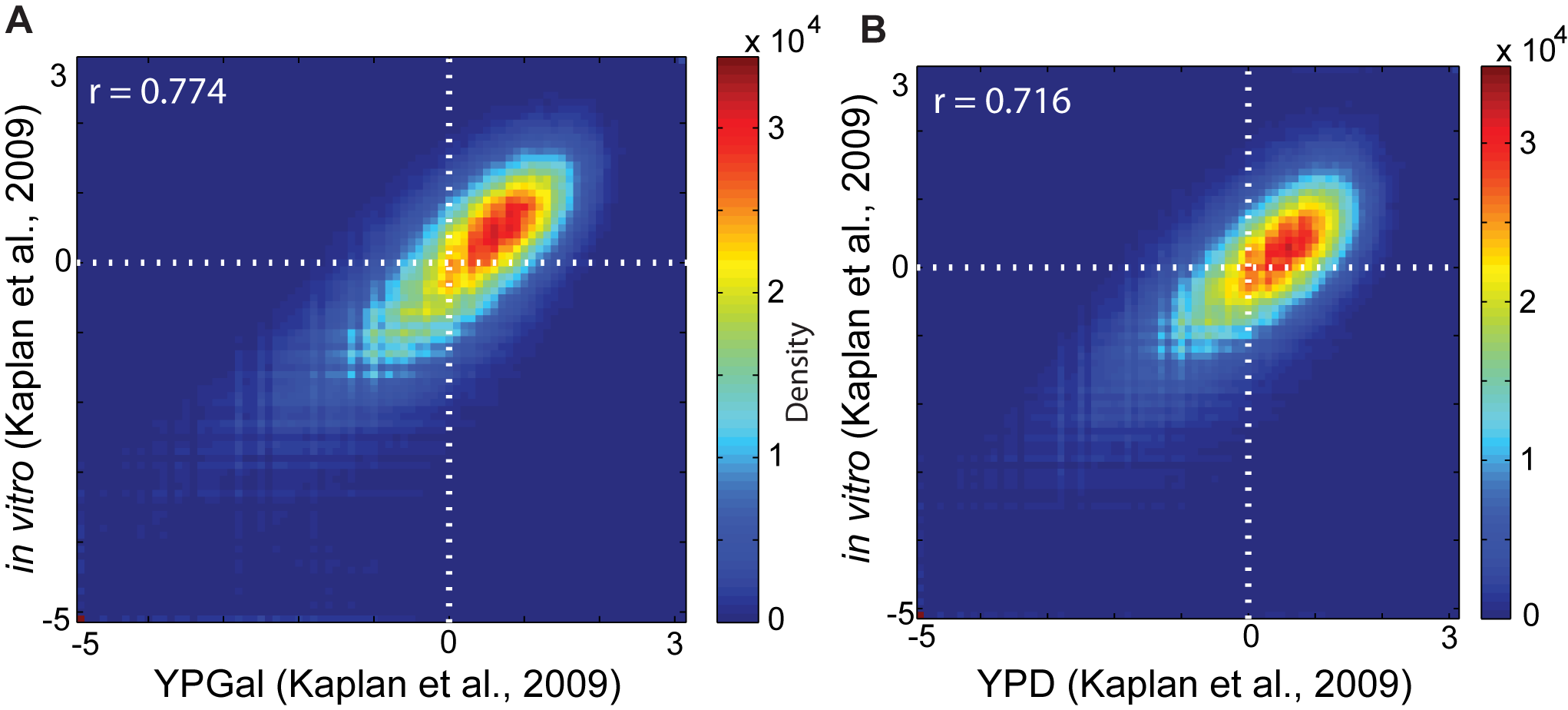

Supplement: Figure S11 — Nucleosome occupancy in galactose-grown cells is more similar to in vitro reconstituted nucleosome occupancy than dextrose-grown cells. (A) In vivo nucleosome occupancy from yeast grown in galactose versus previously published in vitro nucleosome occupancy genome wide [8]. The occupancy values were normalized as in [8]; log2 values are plotted. (B) Same as (A), but for yeast grown in dextrose. (TIF) [file pgen.1002771.s011.tif]

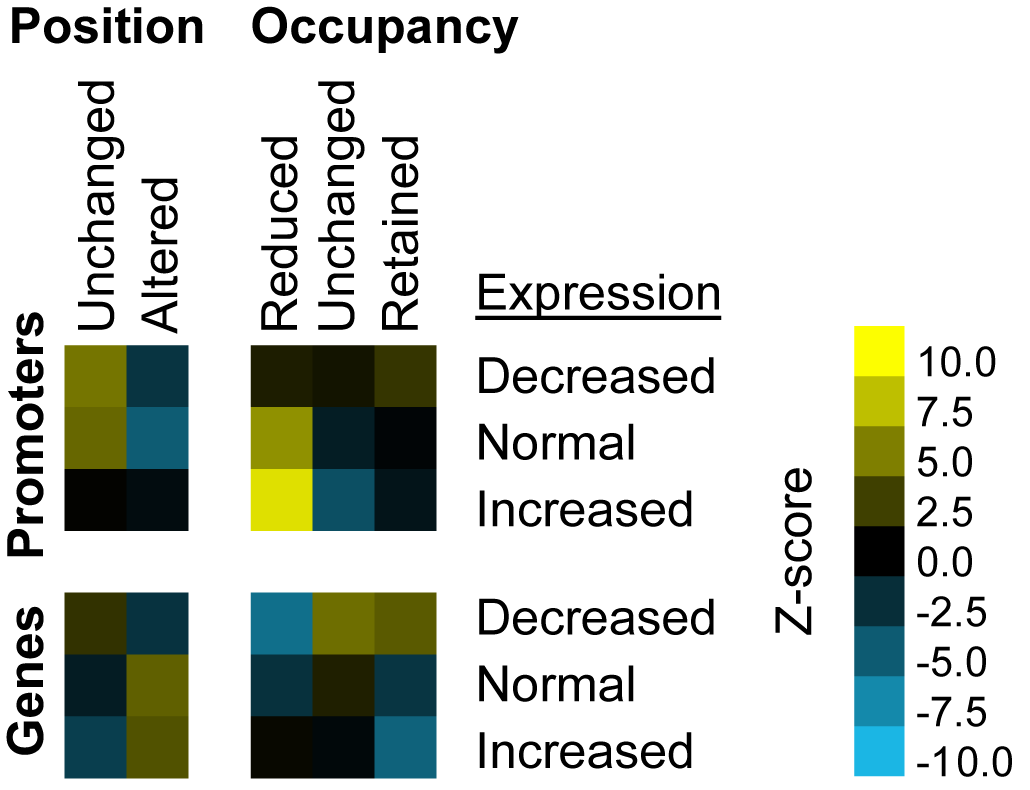

Supplement: Figure S12 — Association of H3-depletion effect categories with the transcriptional response. The relationship between gene expression changes upon histone depletion (rows) and changes in nucleosome occupancy and position (columns) as in Figure 5, except that the colors indicate the Z-score for enrichment rather than the log10 of the p-value. This relationship is shown for promoters, defined as the untranscribed region upstream of a gene, and genes, defined as the transcribed regions from [13]. (TIF) [file pgen.1002771.s012.tif]
