# Supplementary material for: In Vivo Effects of Histone H3 Depletion on Nucleosome Occupancy and Position in Saccharomyces cerevisiae
Source: PLoS Genet. 2012 Jun 21;8(6):e1002771. doi: 10.1371/journal.pgen.1002771 (PMC3380831; doi:10.1371/journal.pgen.1002771)
Supplement: Text S1 — Additional details regarding the western blots, MNase digestions, calculations regarding MNase susceptibility, and details on single-end and paired-end library preparations. (DOCX) [file pgen.1002771.s016.docx]

**Supplemental Methods**

**Western blot analysis**

Cells were grown as stated in the Materials and Methods section. At 0, 1.5, and 3 hours a 1 mL sample was removed and used to prepare whole-cell extract. A total of 1 µg of whole cell extract from each time point was loaded onto a 12% acrylamide gel. Following electrophoresis, the proteins were transferred to nitrocellulose membrane (GE Healtchare Amersham Hybond –ECL, RPN303D). The membrane was blocked in 5% Nonfat Dry Milk (5% NFDM) in 1X TBS with 0.1% Tween (TBS/T) added for 1 hour. The membrane was then cut in half to separate the larger proteins (top; > 40 kDa) from the smaller proteins (bottom; < 40 kDa). The top half of the membrane was incubated overnight with shaking at 4°C with 1:50,000 anti-G6PDH antibody generously provided by Brian Strahl diluted in 5% NFDM in 1X TBS/T. The lower half was incubated with a rabbit polyclonal H3 antibody (abcam ab46765) at a 1:10,000 dilution in 5% NFDM in 1X TBS/T overnight at 4°C with shaking. The membranes were washed individually three times, and then incubated with an anti-rabbit secondary for 1 hour at room temperature at dilutions of 1:15,000 for the top half of the membrane and 1:20,000 for the lower half. The gels were then visualized using ECL (Amersham ECL+). The membranes were scanned using a phosphoimager, and the resulting images were quantified using ImageJ (<http://rsbweb.nih.gov/ij/>).

**MNase digestion**

Cells were fixed with 1% formaldehyde for 15 minutes at room temperature with shaking. Crosslinking was quenched by addition of glycine to a final concentration of 0.125 M and incubation at room temperature for 5 minutes. Cells were then centrifuged at 3000 rpm in a Sorvall RT-34, washed twice with 20 mL of 1 M sorbitol and resuspended in a final volume of 10 mL in spheroplast digestion buffer [1 M sorbitol, 1 mM 2-mercaptoethanol (Fischer Scientific BP176100) and 42,000 U of lyticase (Sigma-Aldrich L2524)]. Cells were rocked at 30°C for 15 minutes, centrifuged as above and washed twice with 10 mL of 1 M sorbitol. Spheroplasts were resuspended at 0.12 g spheroplast/mL of MNase digestion buffer (1 M sorbitol; 50 mM NaCl; 10 mM Tris pH 7.5; 5 mM MgCl_2_; 1 mM CaCl_2_; 0.075% NP-40 with 1 mM 2-mercaptoethanol and 0.5 mM spermidine added immediately before use). Next, 600 µL aliquots of resuspended spheroplasts were digested with a titration of micrococcal nuclease A (Worthington-Biochemical LS004797) ranging from 0 to 50 U for 15 minutes at 37°C. Digestion was stopped with 150 µL of stop buffer (5% SDS, 50 mM EDTA), and then 5 µL of a 20 mg/mL stock solution of Proteinase K (Roche 03 115 879 001) was added. The tube was incubated at 65°C overnight to reverse crosslinking, and DNA was isolated by standard phenol:chloroform extraction (2x) and precipitated using isopropanol. DNA was dried and resuspended in 15 µL of ddH_2_O. To remove RNA, 1 µL of previously boiled 10 mg/mL RNase A (Sigma Aldrich R5503) was added, and the reaction was incubated at 37°C for 30-60 minutes. The DNA was then electrophoresed on a 2% agarose gel, and the band corresponding to a mono-nucleosome band was extracted (Clare ChemicalResearch DR46B Transilluminator).

**MNase susceptibility**

The degree of smearing in MNase digest agarose gels was quantified using ImageJ (<http://rsbweb.nih.gov/ij/index.html>) to measure the intensity of signal from 200 to 250 bp in the lane selected for sequencing (see yellow box, Figure S1F-I). The signal was normalized to the background intensity in the control, MNase-free lane. This was repeated for three replicates, and the average normalized signal between bands was compared between wildtype and H3 shutoff cells. The signal at 200-250 bp was found to be significantly higher at 3 hours compared to 0 hours for the H3 shutoff strain (p < 0.05).

**Single-end sequencing preparation**

Single-end samples were prepared using a modified version of the Illumina Solexa preparation protocol. DNA was blunted using the END-IT DNA Repair Kit (Epicentre ER0720) by incubating 50 ng of mononucleosome DNA with 5 µL 10x buffer, 5 µL dNTP mix, 5 µL ATP solution, and 1 µL enzyme mix in a 50 µL reaction for 60 minutes at room temperature. DNA was recovered using the QIAQuick Gel Extraction kit (Qiagen 28704) with 50 µL ddH_2_O and 400 µL of QG buffer. DNA was eluted using two 15 µL elutions and then adenylated using 1 µL of NEB exo- klenow (M212M), 5 µL Klenow buffer, and 1 µL 10mM dATP in a 50 µL reaction for 60 minutes at room temperature. DNA was isolated using a QIAquick MinElute kit (Qiagen 28006) and eluted in 10 µL of EB. Adapters were ligated using NEB Quick T4 DNA Ligase (M2200S) with a 10:1 dilution of adapters provided by the UNC High Throughput Sequencing Facility (UNC HTSF) in a 50 µL reaction containing 10 µL of DNA, 25 µL 2X Quick Ligation Buffer, 2 µL adapters, and 1 µL DNA ligase for 25 minutes at room temperature. DNA was purified using a QIAQuick MinElute column and eluted in 10 µL of EB. DNA was amplified using Stratagene PfuUltra II Fusion HS DNA Polymerase (Stratagene 600670) using 2 µL DNA, 2 µL Solexa single-end PCR primers, 10 µL 10X PfuUltra II buffer, 10 µL 2.5 mM dNTP, and 1 µL of Pfu Ultra II DNA polymerase in a 100 µL reaction with the following cycling conditions: 95°C for 1 minute; 18 cycles of 95°C for 50 seconds, 65°C for 60 seconds, 72°C for 30 seconds; 72°C for 5 minutes; 4°C hold. DNA was purified using the QIAQuick MinElute kit in 10 µL of buffer EB and run out on a 2% agarose gel. The band corresponding to mononucleosome + adapters (approximately 250 base pairs) was cut out on the dark reader, gel extracted using the QIAQuick Gel Extraction kit, and submitted to UNC HTSF for sequencing.

Two H3 depletion and one wildtype replicate(s) were submitted for single-end sequencing.

**Paired-end sequencing preparation**

Paired-end samples were prepared using a modified version of the Illumina Solexa preparation protocol provided by the UNC HTSF (v 2.5). End-repair was performed using 50 ng of gel extracted, mononucleosomal DNA in 30 µL of H_2_O, 9.8 µL of water, 5 µL of 10x NEB T4 Ligation Buffer with ATP, 2 µL of 10 mM dNTP mix, 1 µL NEB PNK, 1 µL of NEB Klenow at 1 U/ µL, and 1.2 µL of NEB T4 DNA polymerase (NEB, M0203S, M0202 and M0210S). The reaction was incubated at room temperature (RT) for 30 minutes and purified using Qiagen MinElute columns (final elution in 25 µL of Buffer EB). The end-repaired DNA was then adenylated by adding 7 µL of H_2_O, 5 µL of Klenow buffer, 10 µL of 1 mM dATP, and 3 µL of Klenow 3’ to 5’ exo- (5 U/ µL; NEB, M0212S). The reaction was incubated at 37°C for 30 minutes before being cleaned up with a Qiagen MinElute column (final elution volume 16 µL of Buffer EB). The Illumina Paired-End adapters were provided by the UNC HTSF, and 1 µL of 1:10 diluted adapters were annealed in a reaction with 20 µL of 2X Quick Ligation buffer, 1 µL of 1.5 µM multiplex adapters, and 1 µL of Quick DNA Ligase (500 U/µL; NEB, M2200S). The samples were incubated at RT for 25 minutes and then purified twice with Ampure XP (Beckman Coulter, #A63881) beads according to the manufacturer’s protocol after adding 10 µ L of Buffer EB to the ligation reaction. For the first purification, 45 µL of beads and a 50 µL elution was used, while the second purification used 40 µL of Ampure XP beads with a 36 µL elution.

Half of the ligated DNA was used for a multiplex PCR amplification reaction in a 50 µL volume [18 µL of ligated DNA, 17 µL of dH_2_O, 10 µL of 5X Phusion Buffer HF, 1 µL of 25 µM PCR primer InPE 1.1 (UNC HTSF), 1 µL of 0.5 µM InPE PCR primer 2.0 (UNC HTSF), 1 µL of PCR Primer Index # (UNC HTSF, see sequences below), 1.5 µL of 10 mM dNTP mix, and 0.5 µL of Phusion polymerase (NEB, F-530S)]. The multiplexing adapter primers used were Index 4 (5’-TGACCA -3’), 5 (5’- ACAGTG -3’) or 6 (5’-GCCAAT-3’). The amplification protocol was 98°C for 30 seconds, followed by 15 rounds of 98°C for 10 seconds, 65°C for 30 seconds, 72°C for 30 seconds with a final incubation at 72°C prior to holding at 4°C. The DNA was then purified using 40 µL of Ampure XP beads and eluted into 20 µL of Buffer EB. The DNA concentration was checked by Qubit prior to submission for sequencing.

Two H3 depletion replicates and two wildtype replicates were submitted for paired-end sequencing. Samples prepared for RNA-seq were prepared as above but submitted for single-end sequencing.
